# Supplementary material for: APOBEC shapes tumor evolution and age at onset of lung cancer in smokers
Source: bioRxiv. 2024 Apr 3:2024.04.02.587805. Preprint. [Version 2] doi: 10.1101/2024.04.02.587805 (PMC11014539; doi:10.1101/2024.04.02.587805)

Extended Data Fig. 1

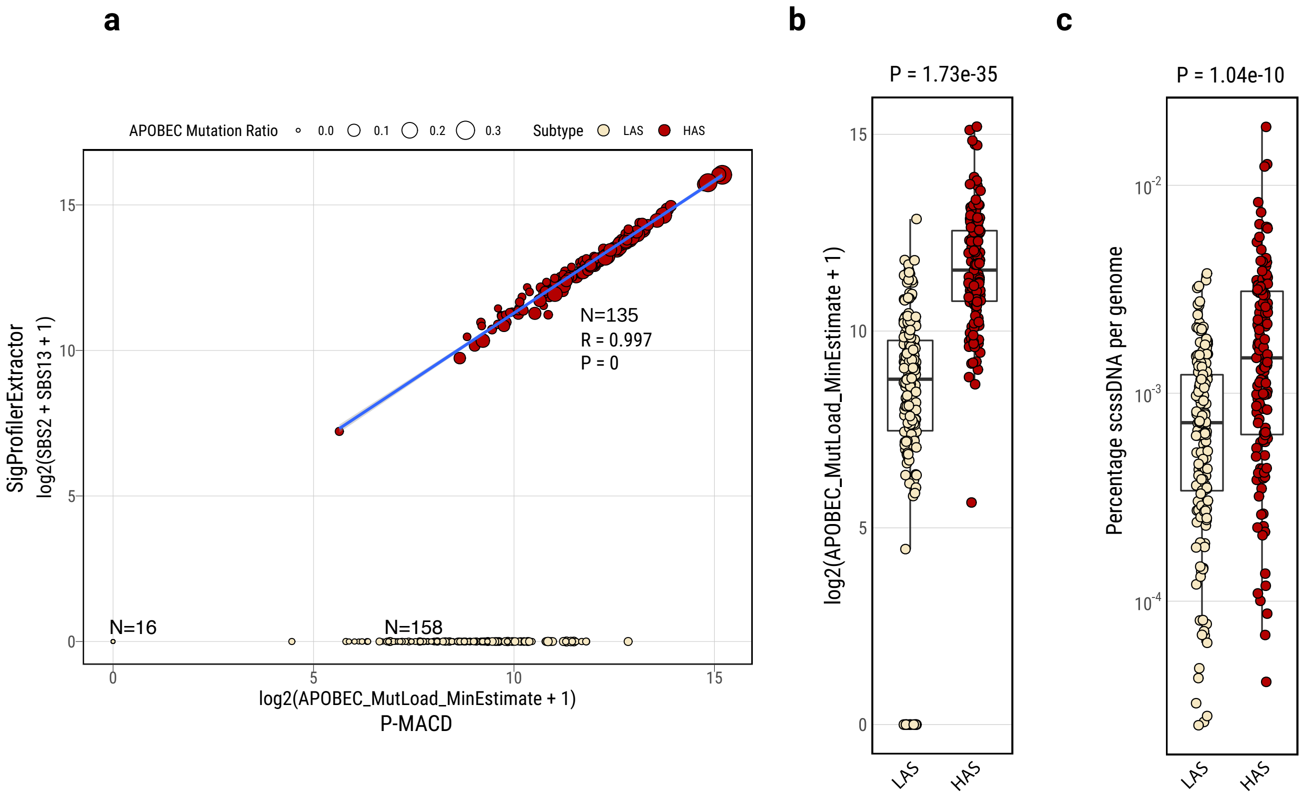

Extended Data Fig. 2

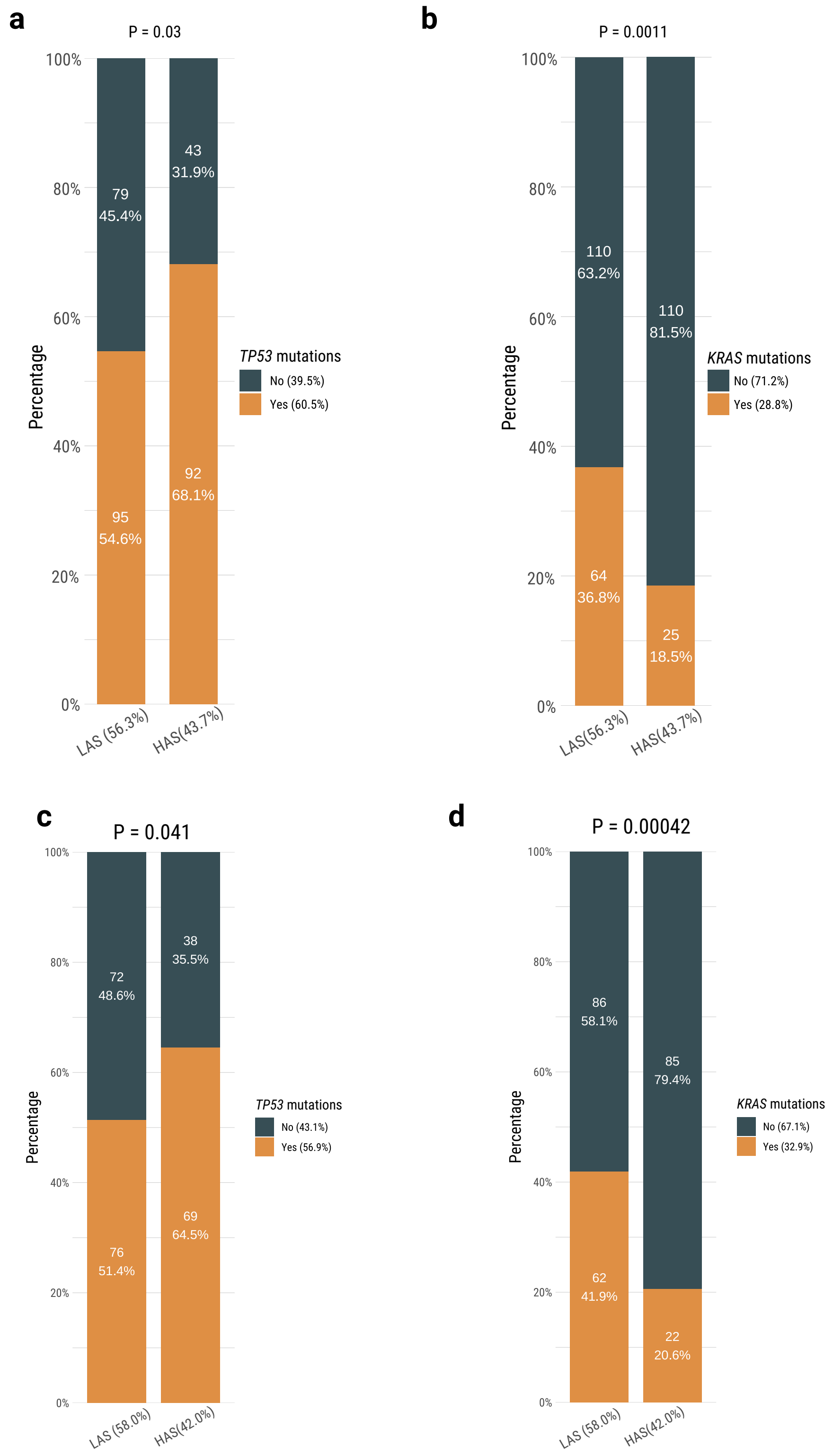

Extended Data Fig. 3

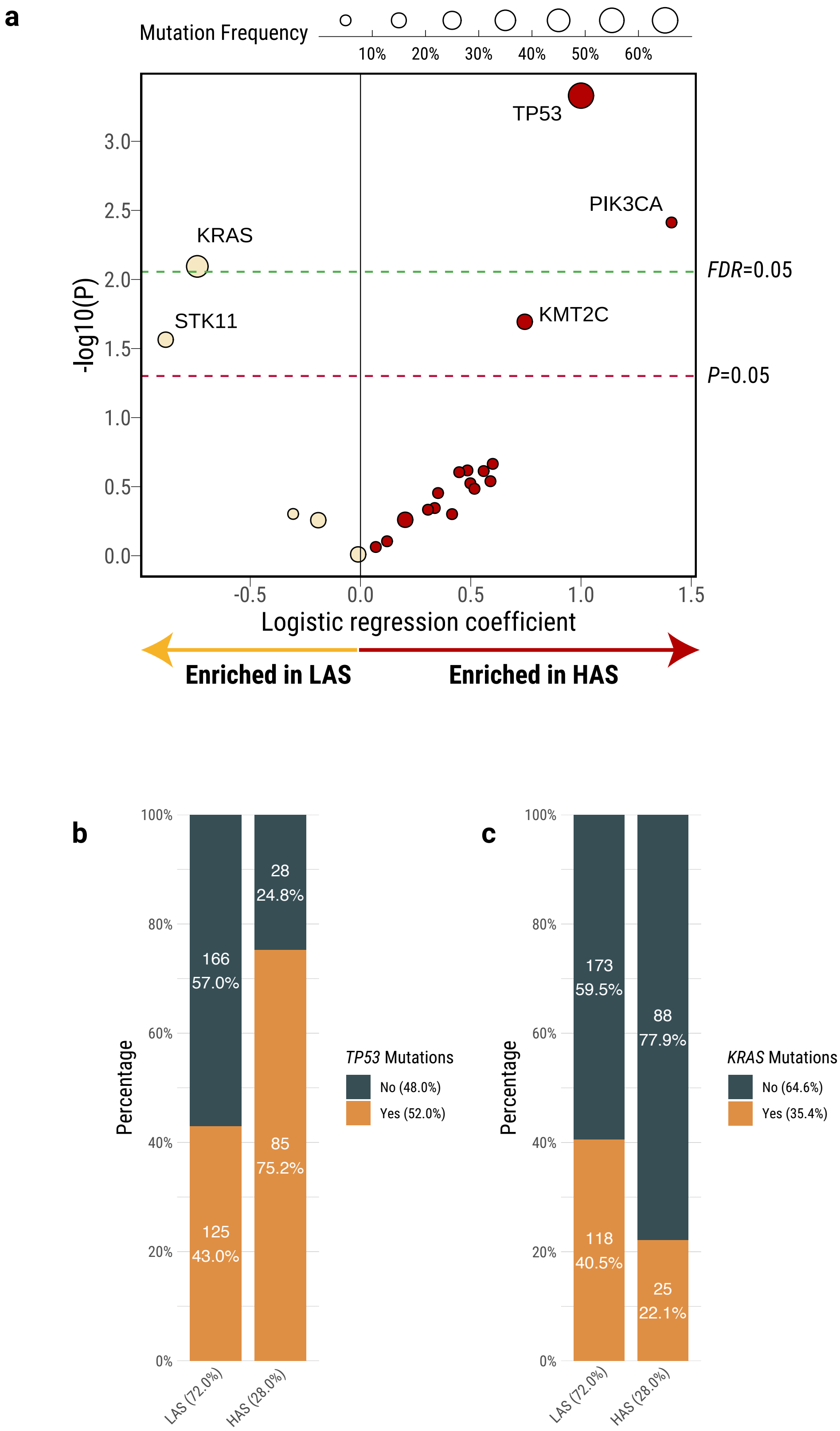

Extended Data Fig. 4

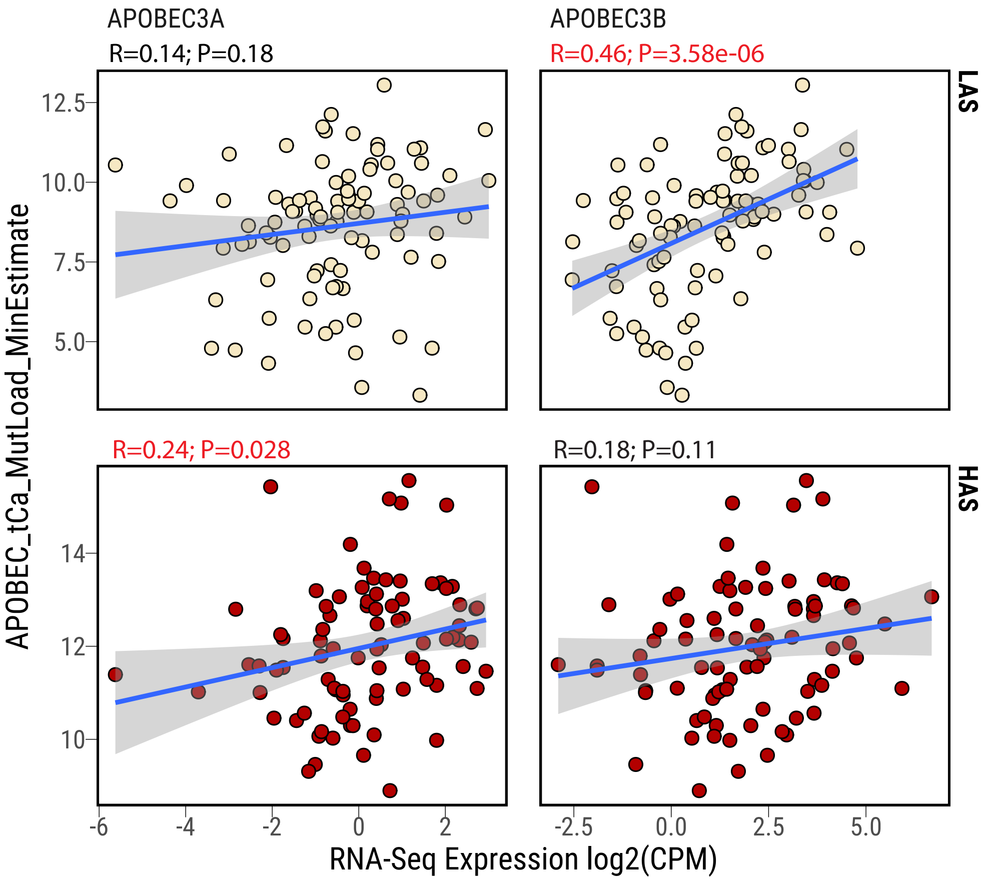

Extended Data Fig. 5

a

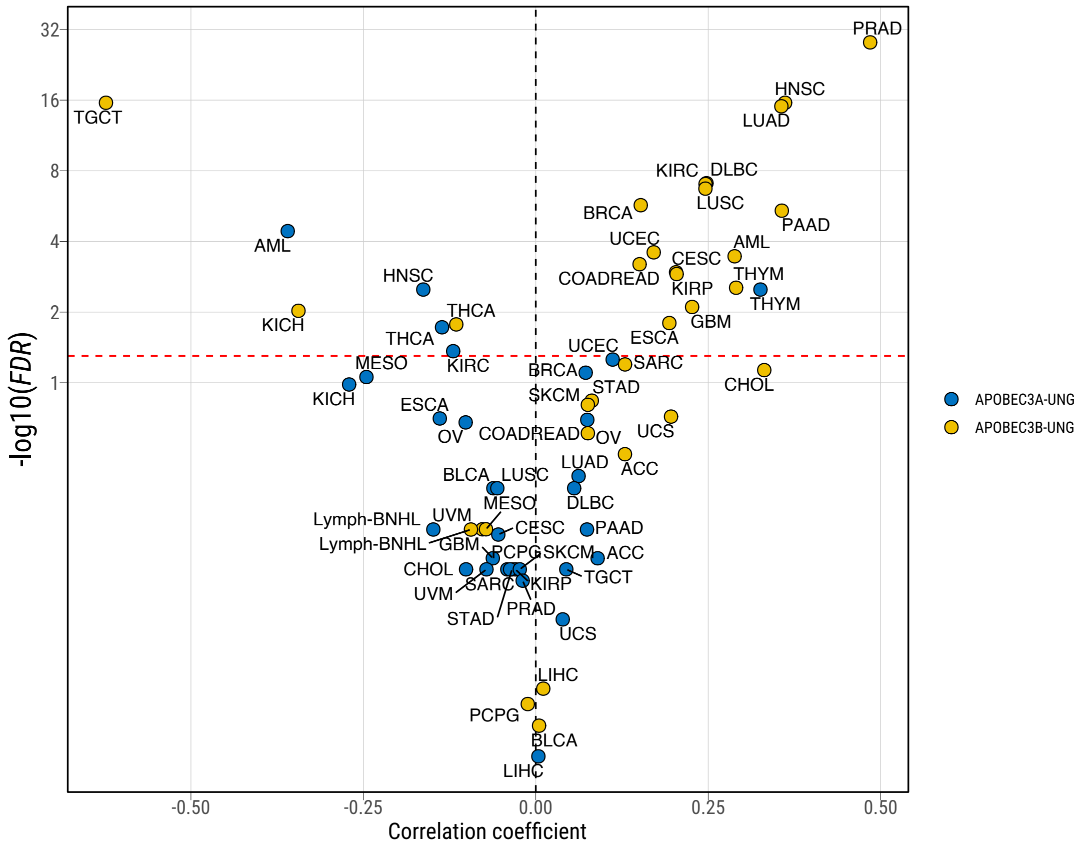

b

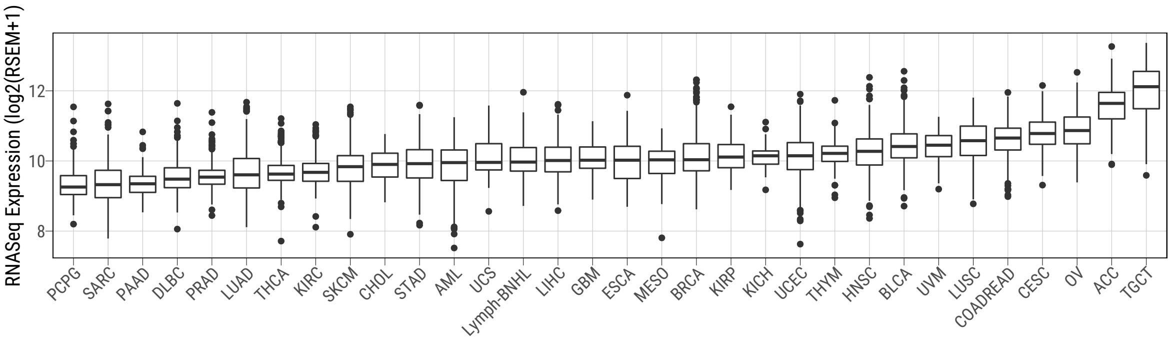

Extended Data Fig. 6

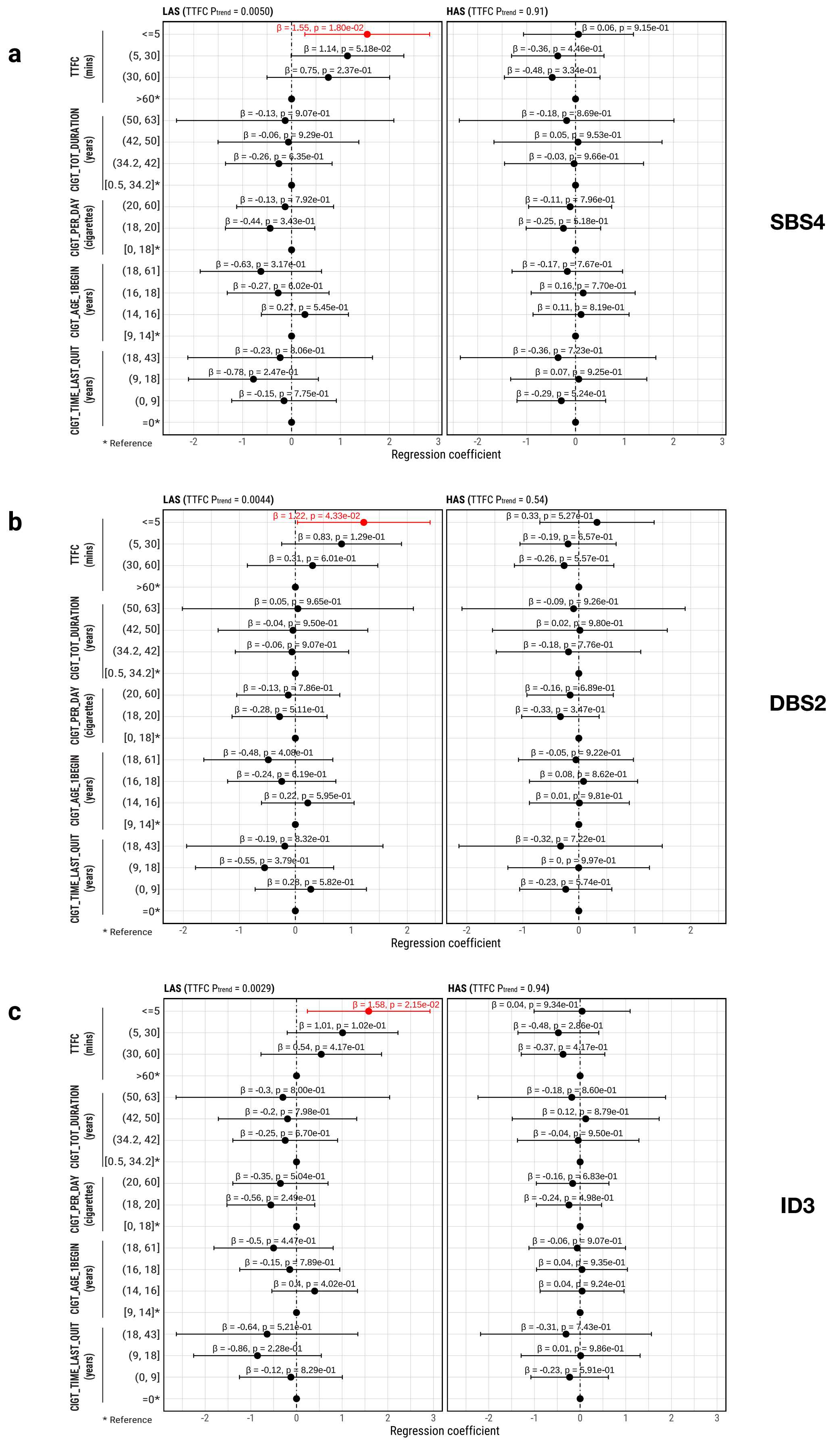

Extended Data Fig. 7

a

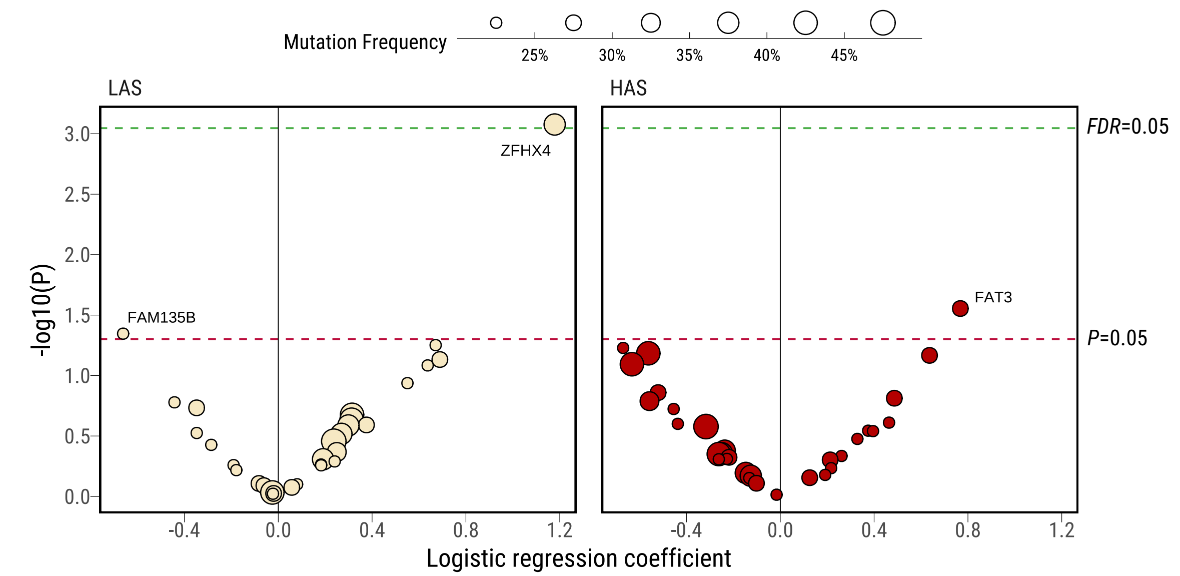

b

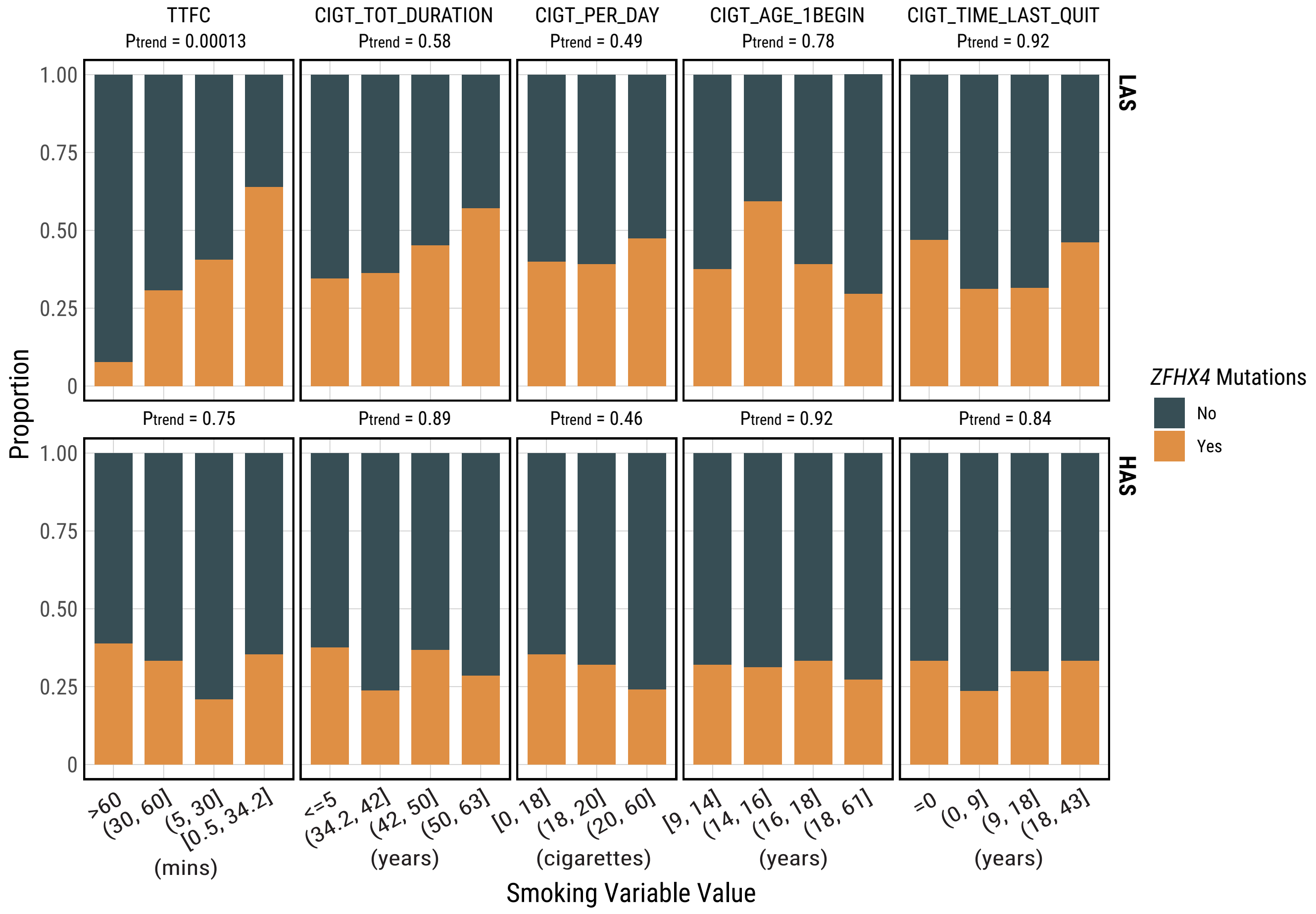

Extended Data Fig. 8

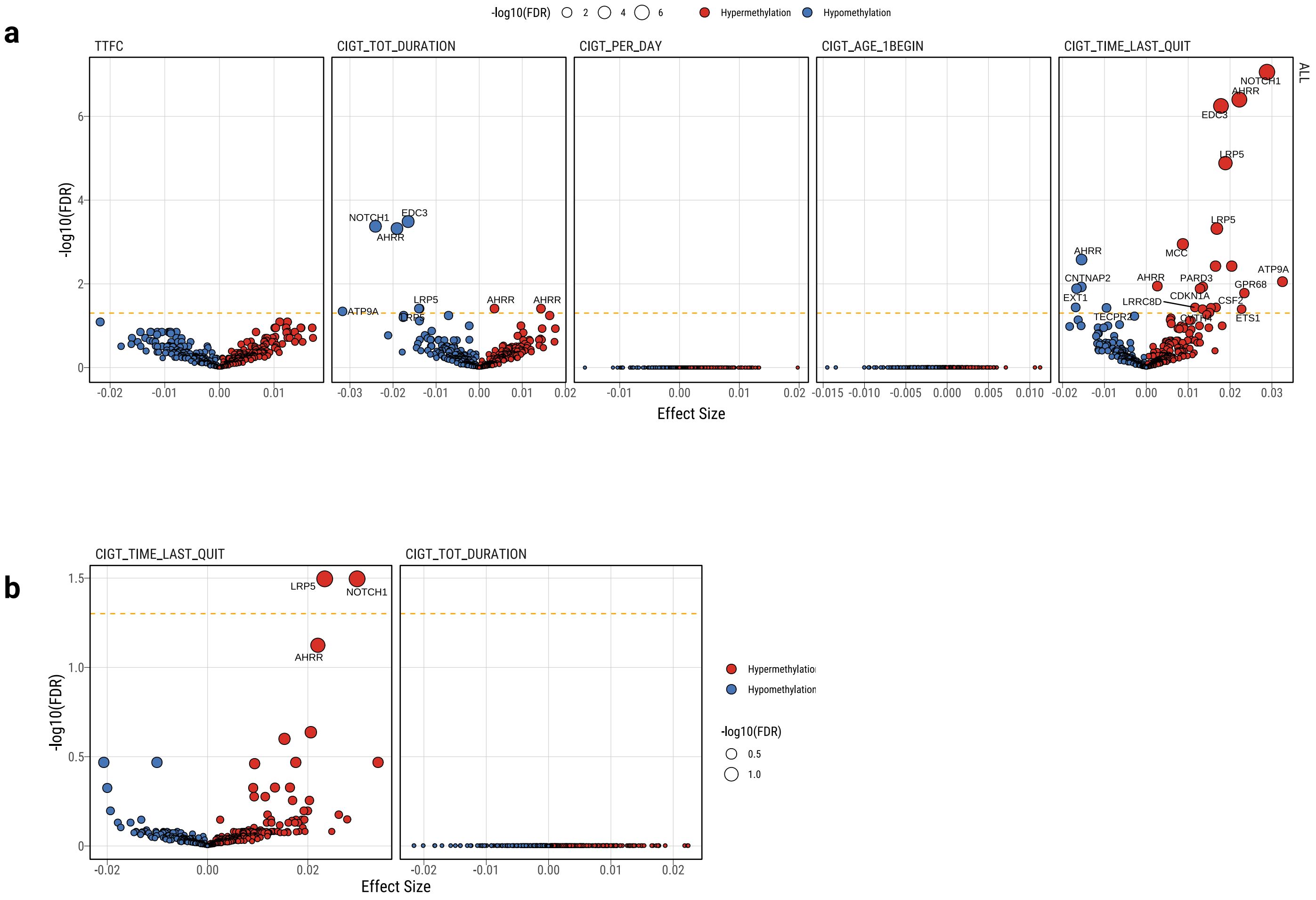

Supplement: Supplement 1 — Extended Data Fig. 1: Characterization of APOBEC mutagenesis based on P-MACD approach between LAS and HAS tumors. a, Comparison of APOBEC mutational signatures detection between the NMF-based SigProfileExtractor approach and the mutational pattern-based P-MACD approach. APOBEC mutation detection is almost identical between the two approaches in 135 tumors, while P-MACD identifies APOBEC mutations below the detection limit of SigProfileExtractor in 158 tumors. b,c, Comparisons of minimal estimated APOBEC mutation load (b) and percentage of hypermutable strand-coordinated ssDNA (scssDNA) per genome due to A3A mutagenesis (c) between LAS and HAS tumors. Extended Data Fig. 2: a-d, Enrichment of TP53 and KRAS mutations between LAS and HAS tumors in all samples (a,b) and LUAD samples only (c-d). P-values of the Fisher’s exact test are shown on the top of each barplot. Extended Data Fig. 3: a, Logistic regression analysis between tumor subtypes and nonsynonymous mutation status of driver genes in TCGA LUAD dataset, adjusting for the following covariates: age, sex, histology, TMB, and tumor purity. The significance levels P<0.05 (red) and FDR<0.05 (green) are indicated by the dashed lines. Bar plots show TP53 (b) and KRAS (c) mutation enrichment between LAS and HAS tumors. Extended Data Fig. 4: Correlation between minimal estimated APOBEC TCA mutational load from P-MACD and gene expression of APOBEC3A and APOBEC3B, stratified by LAS and HAS tumors. Pearson correlation coefficients and P-values are labeled above each plot and in red ink if P<0.05. Extended Data Fig. 5: Validation of gene expression correlation between UNG and APOBEC3A and APOBEC3B in all TCGA cancer types. a, Volcano plot shows the correlations between UNG and APOBEC3A (blue) and between UNG and APOBEC3B (yellow). The suggested significance level FDR=0.05 is shown as a dashed red line. b, UNG gene expression level across all TCGA cancer types sorted by the median UNG expression. Cancer type abbreviations fro [file media-1.pdf]
