# Supplementary material for: APOBEC shapes tumor evolution and age at onset of lung cancer in smokers
Source: bioRxiv. 2024 Apr 3:2024.04.02.587805. Preprint. [Version 2] doi: 10.1101/2024.04.02.587805 (PMC11014539; doi:10.1101/2024.04.02.587805)

Supplementary Fig. 1

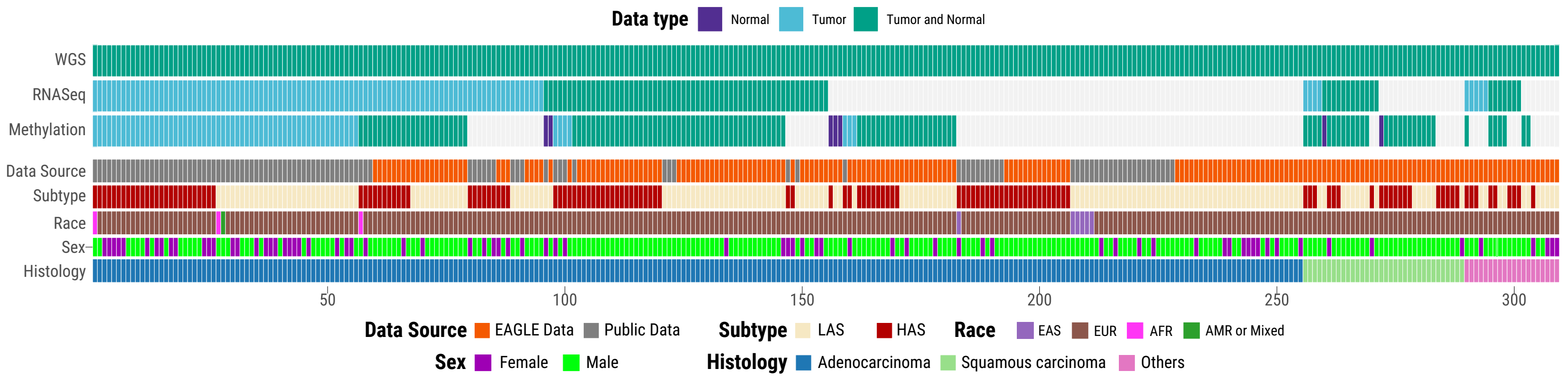

Supplementary Fig. 2

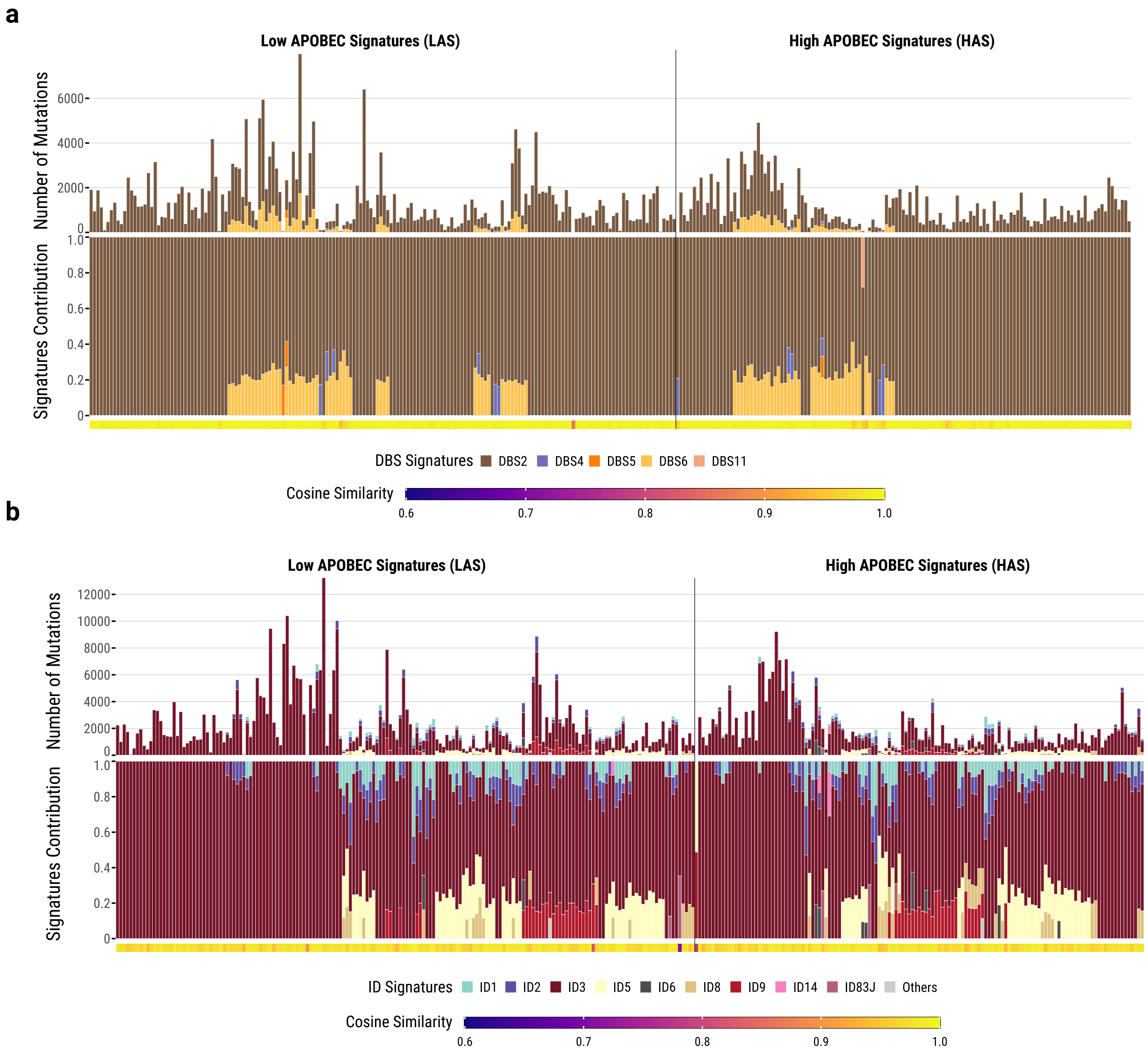

Supplementary Fig. 3

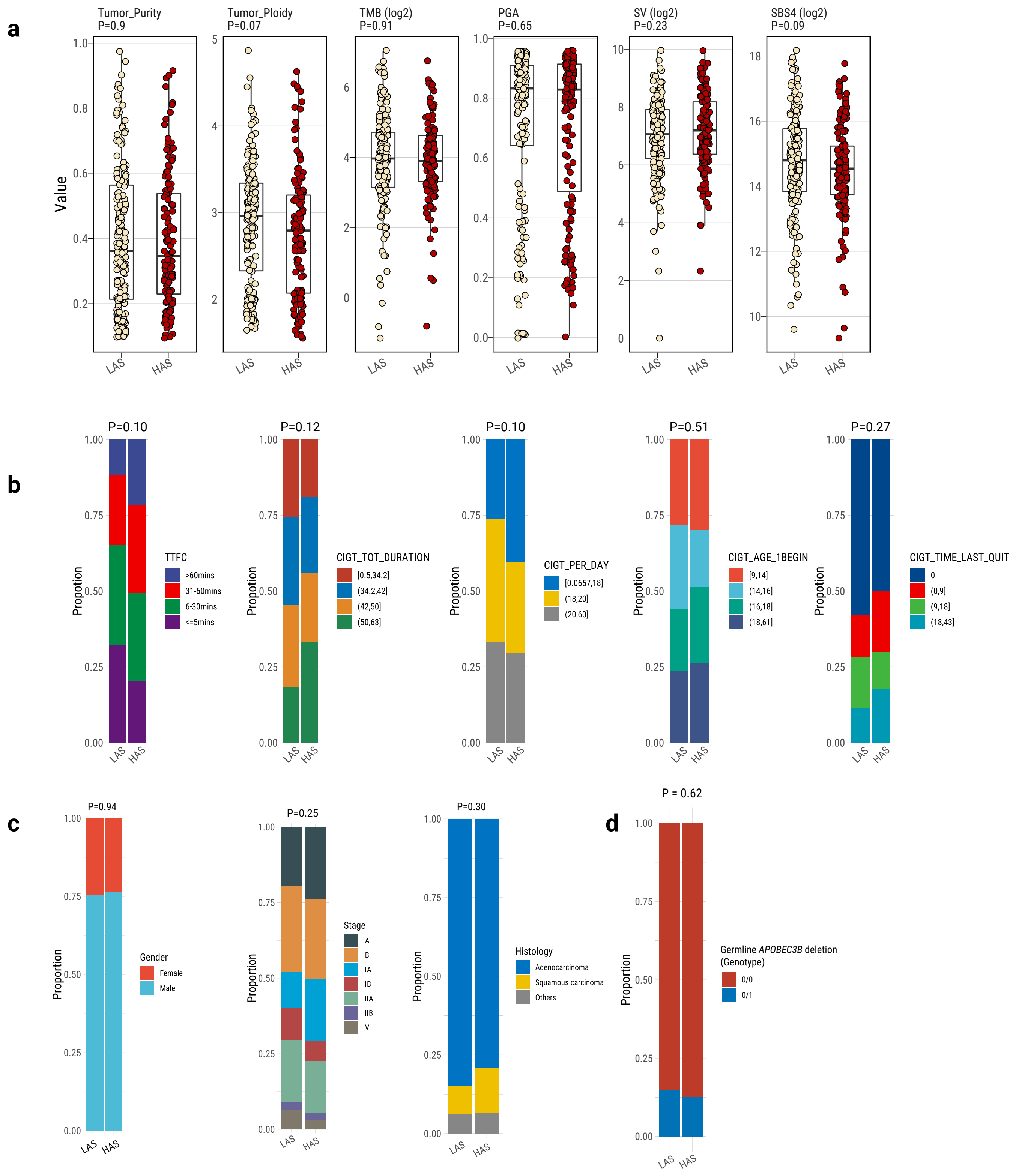

Supplementary Fig. 4

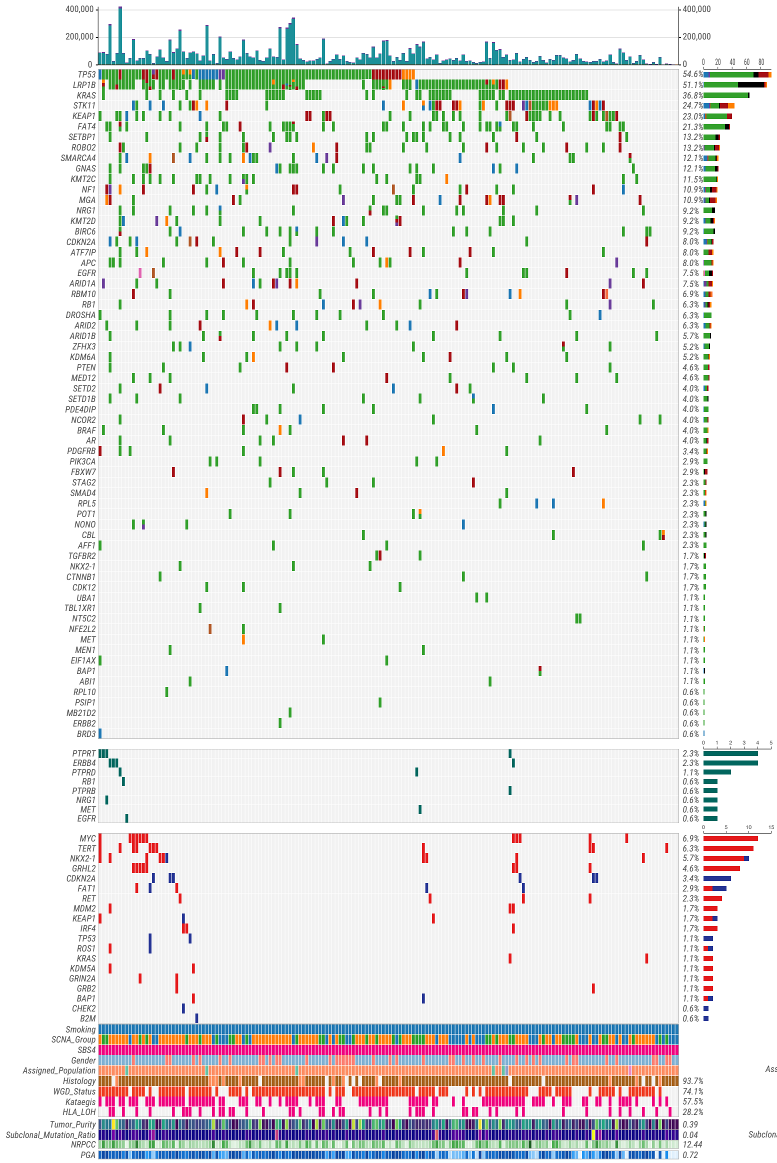

LAS

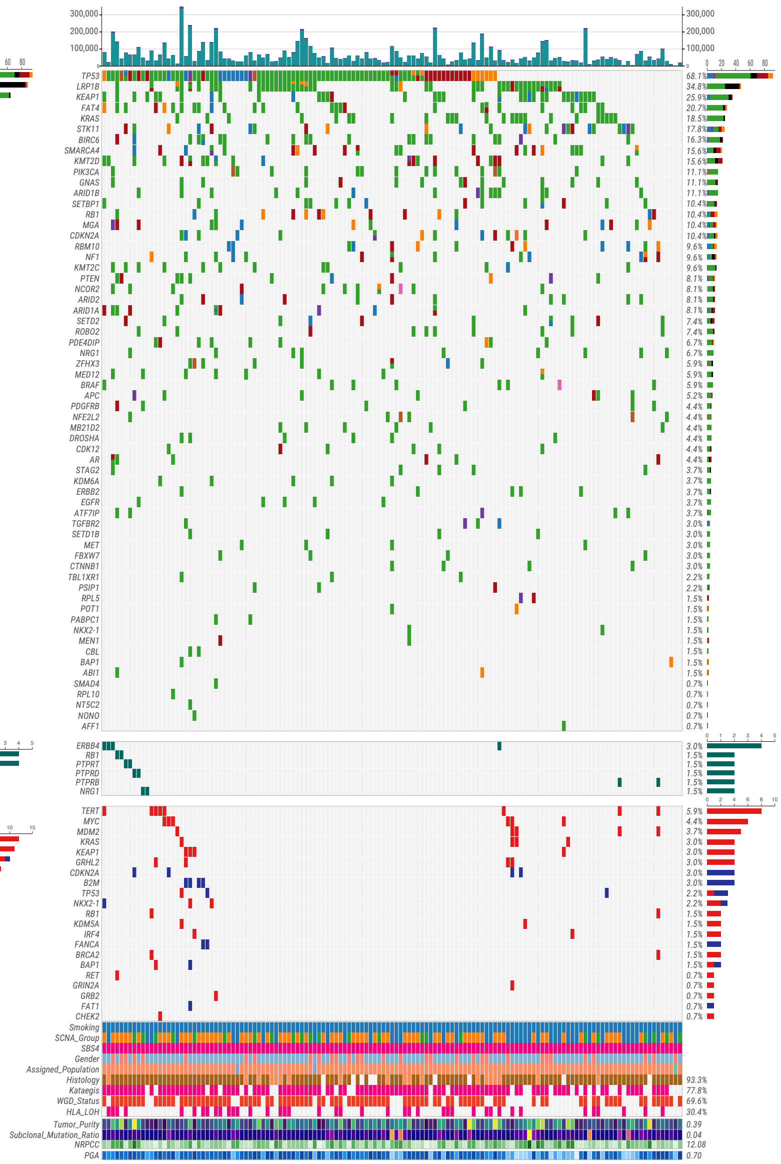

HAS

Supplementary Fig. 5

a

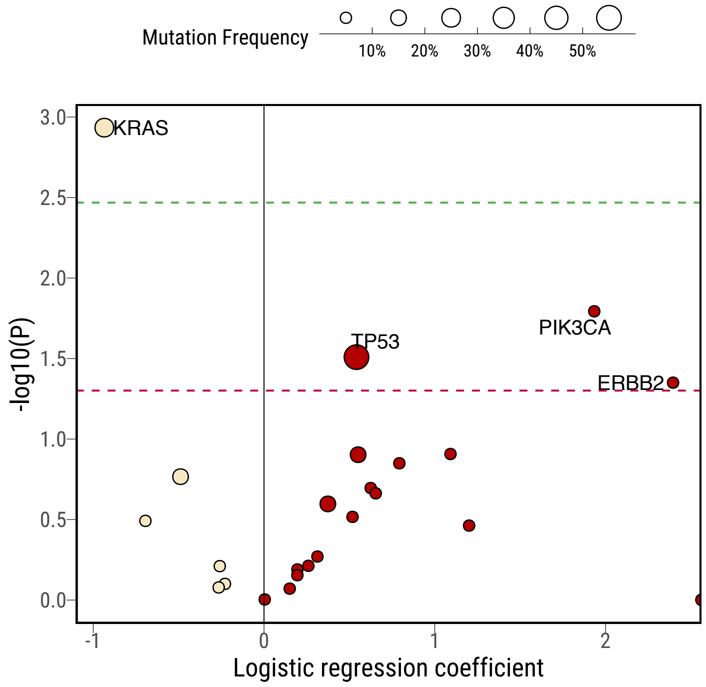

b

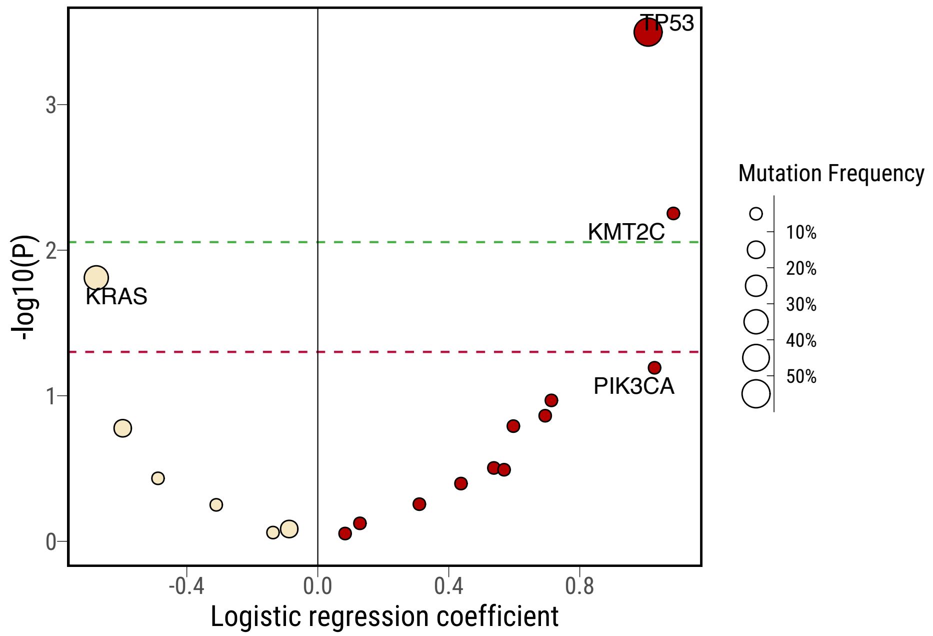

Supplementary Fig. 6

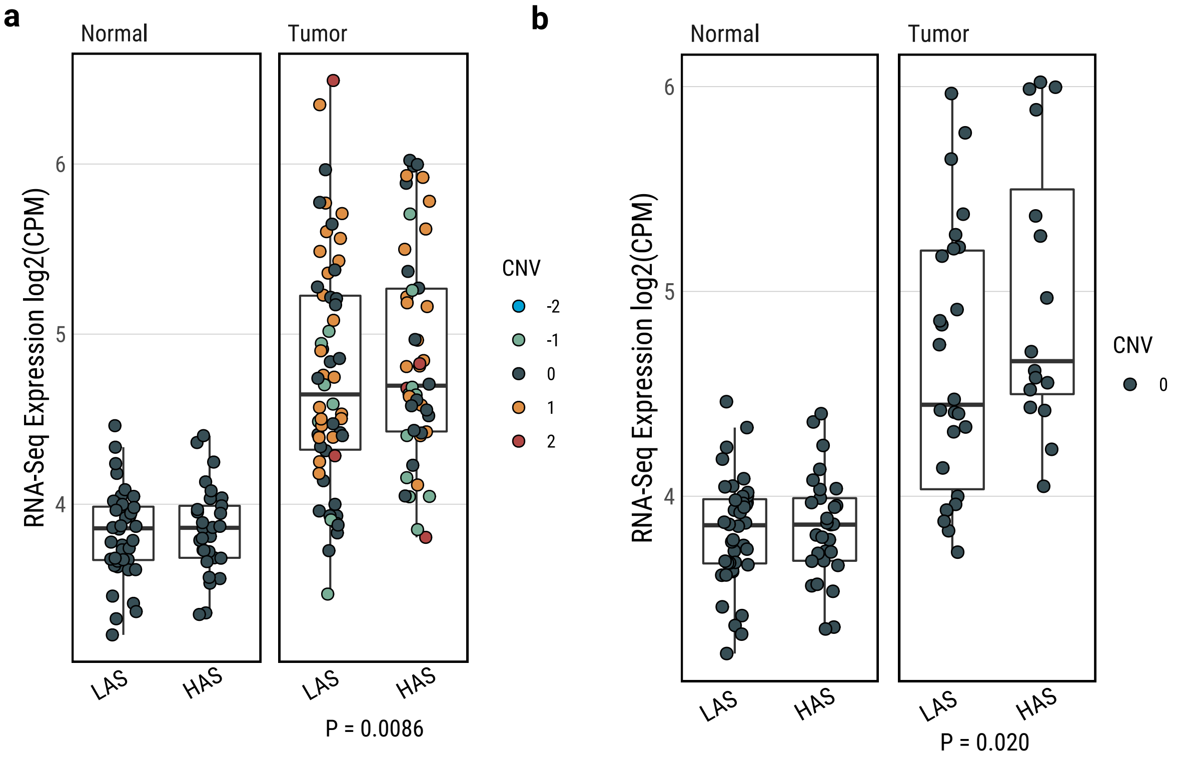

Supplementary Fig. 7

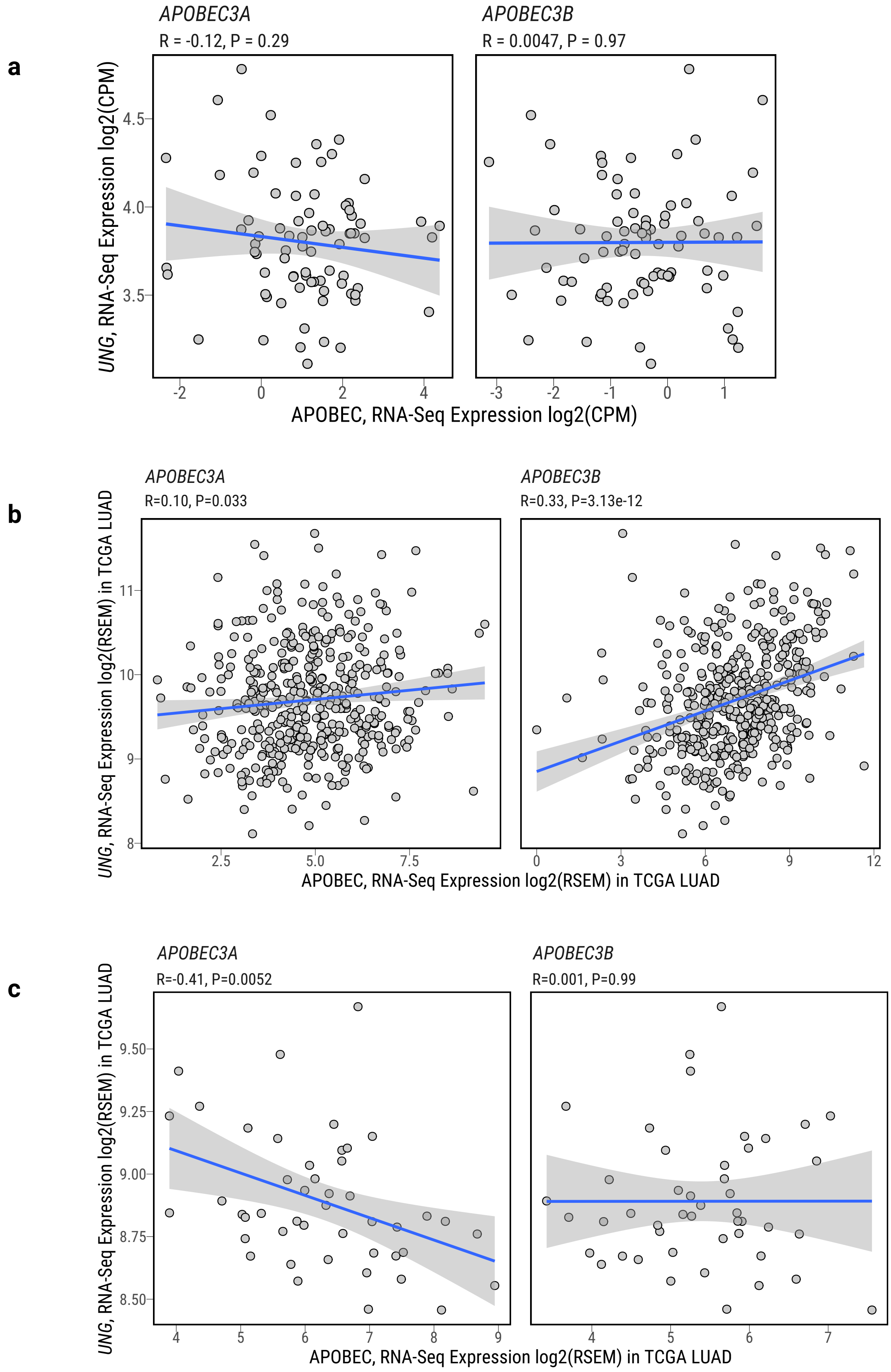

Supplementary Fig. 8

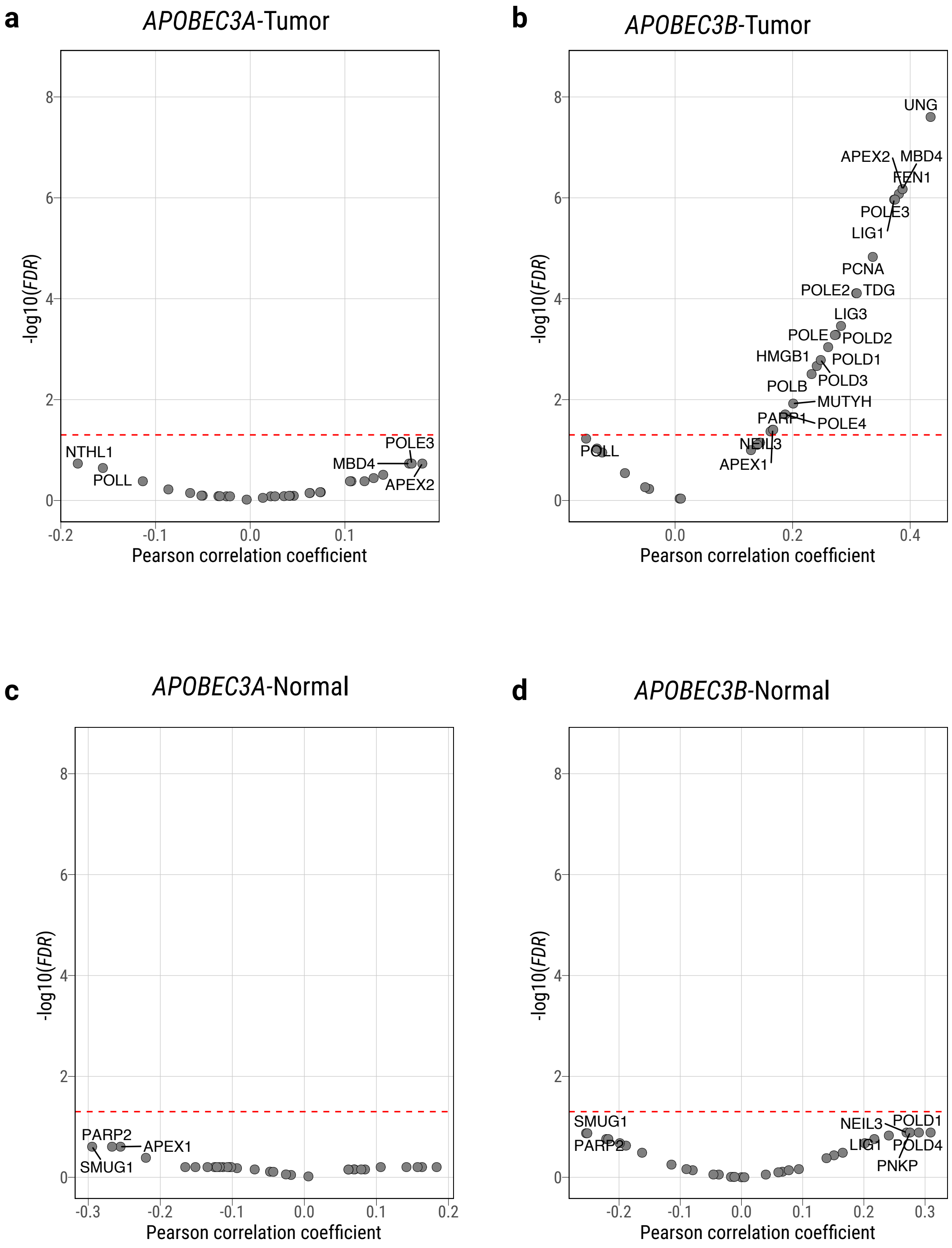

Supplementary Fig. 9

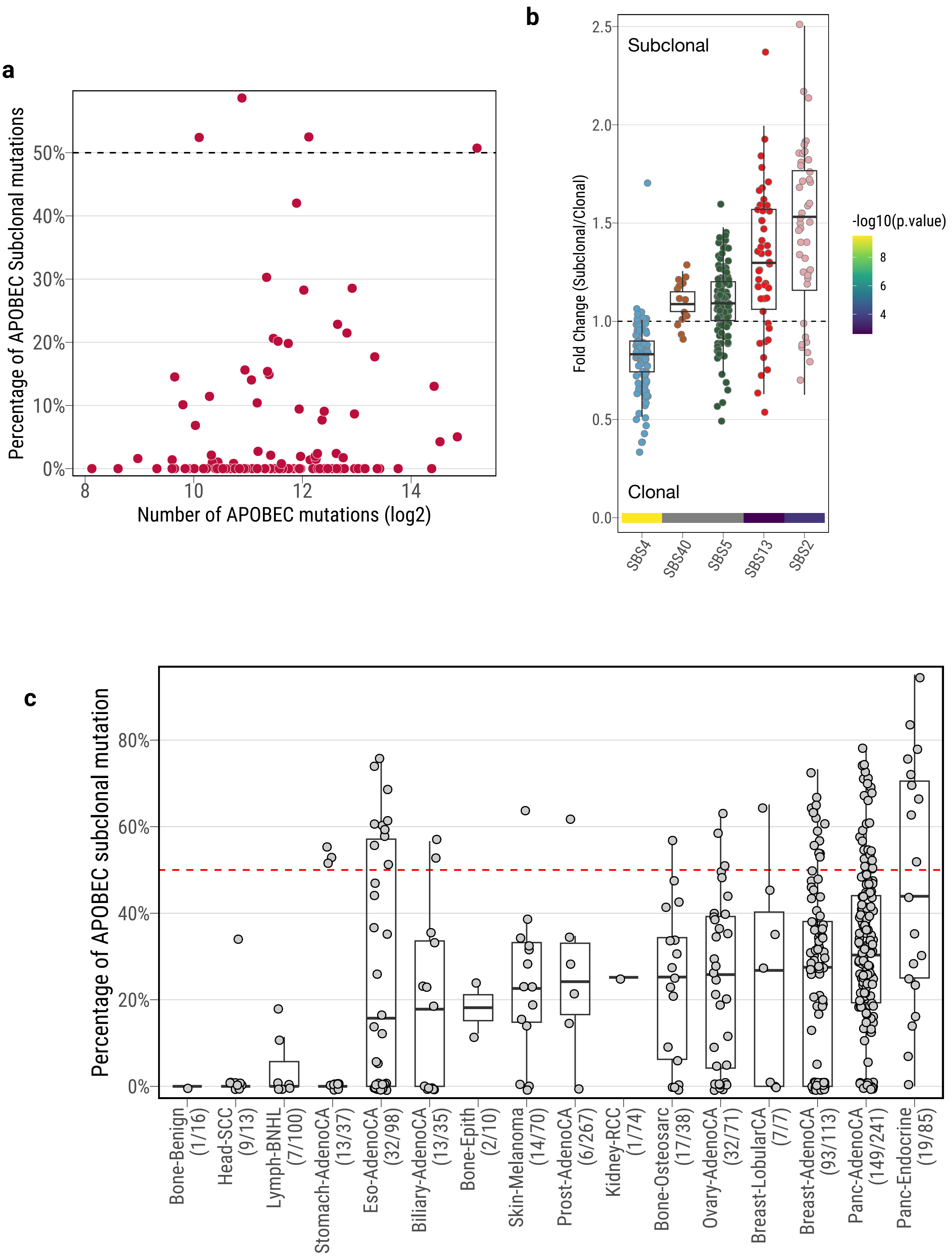

Supplementary Fig. 10

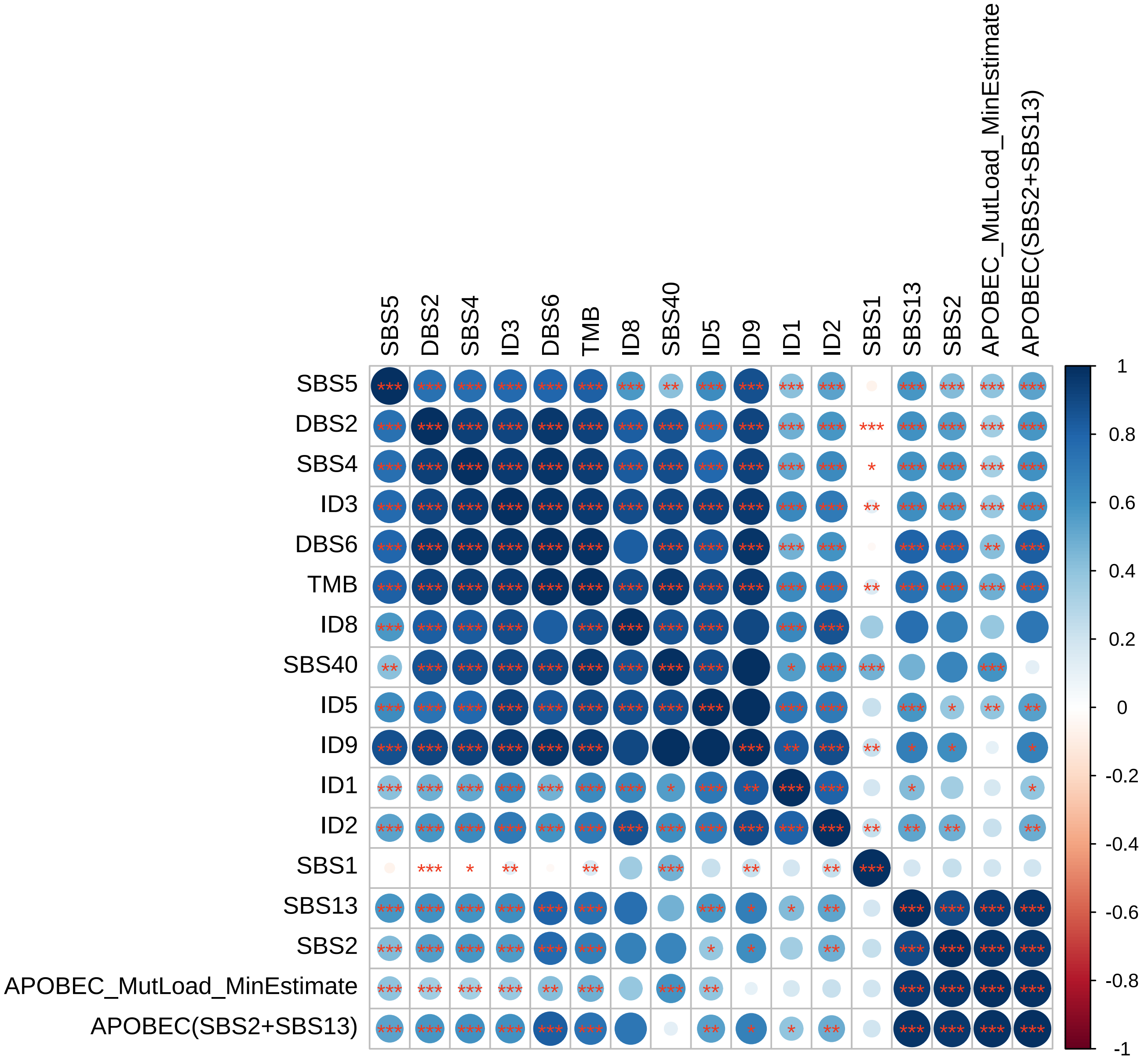

Supplementary Fig. 11

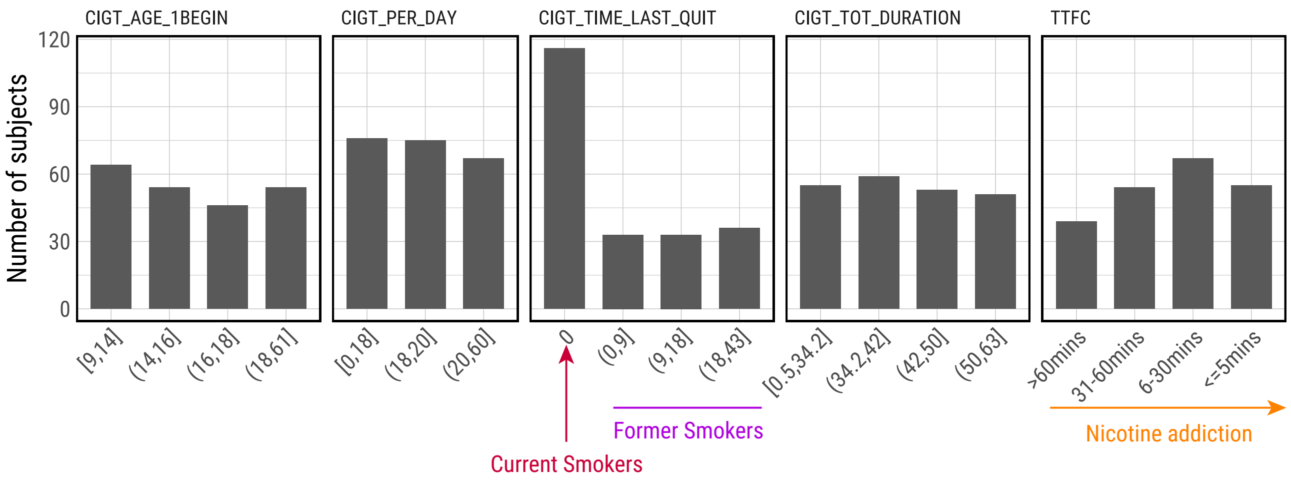

CIGT_AGE_1BEGIN: Age (in years) when subjects started smoking cigarettes regularly for the first time.
CIGT_PER_DAY: Average intensity of cigarette smoking, measured as the number of cigarettes per day.
CIGT_TIME_LAST_QUIT: Number of years since the subject quitted smoking cigarettes (0 means current smokers).
CIGT_TOT_DURATION: Total period (in years) during which the subject smoked cigarettes regularly.
TTFC: Time to first cigarette in the morning (from the first question of the Fagerstrom test for nicotine dependence: 'How soon after you wake up do you smoke your first cigarette?')

Supplementary Fig. 12

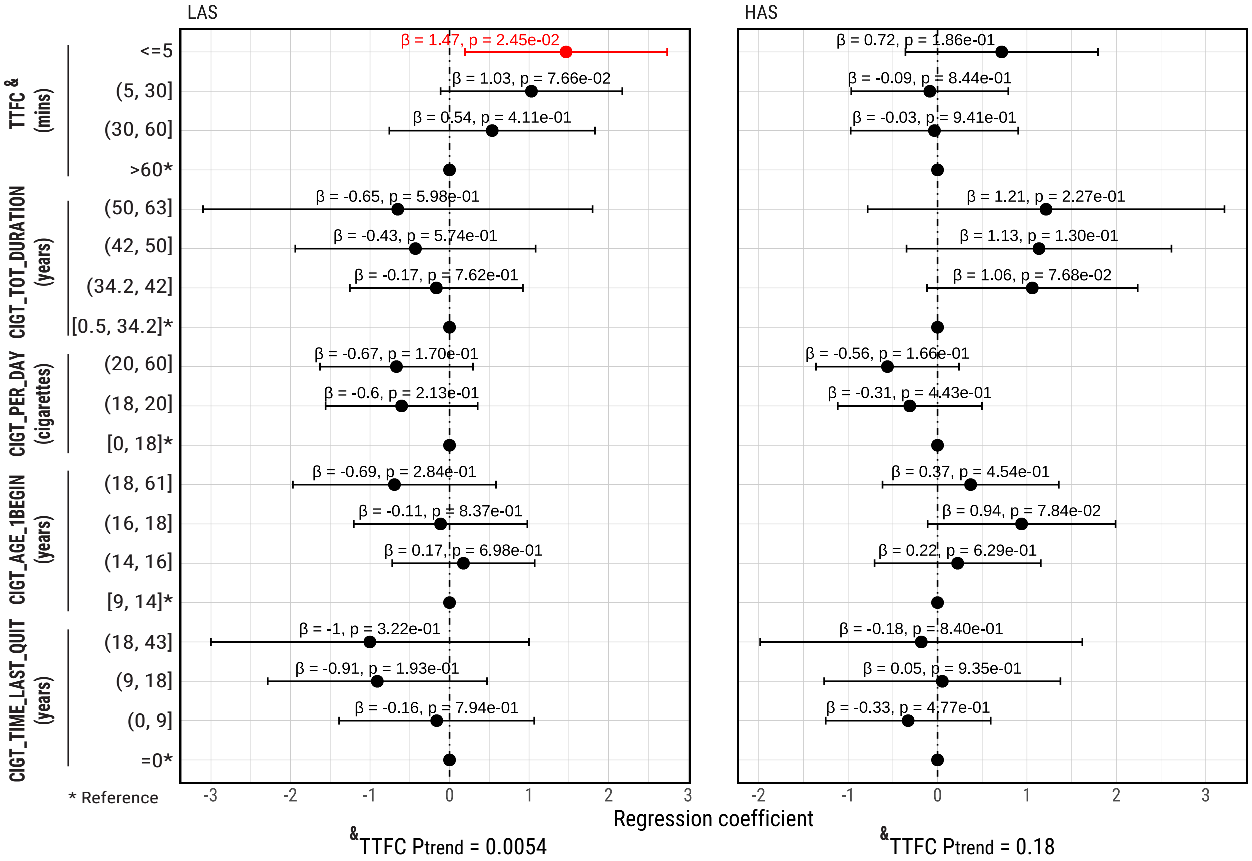

Supplementary Fig. 13

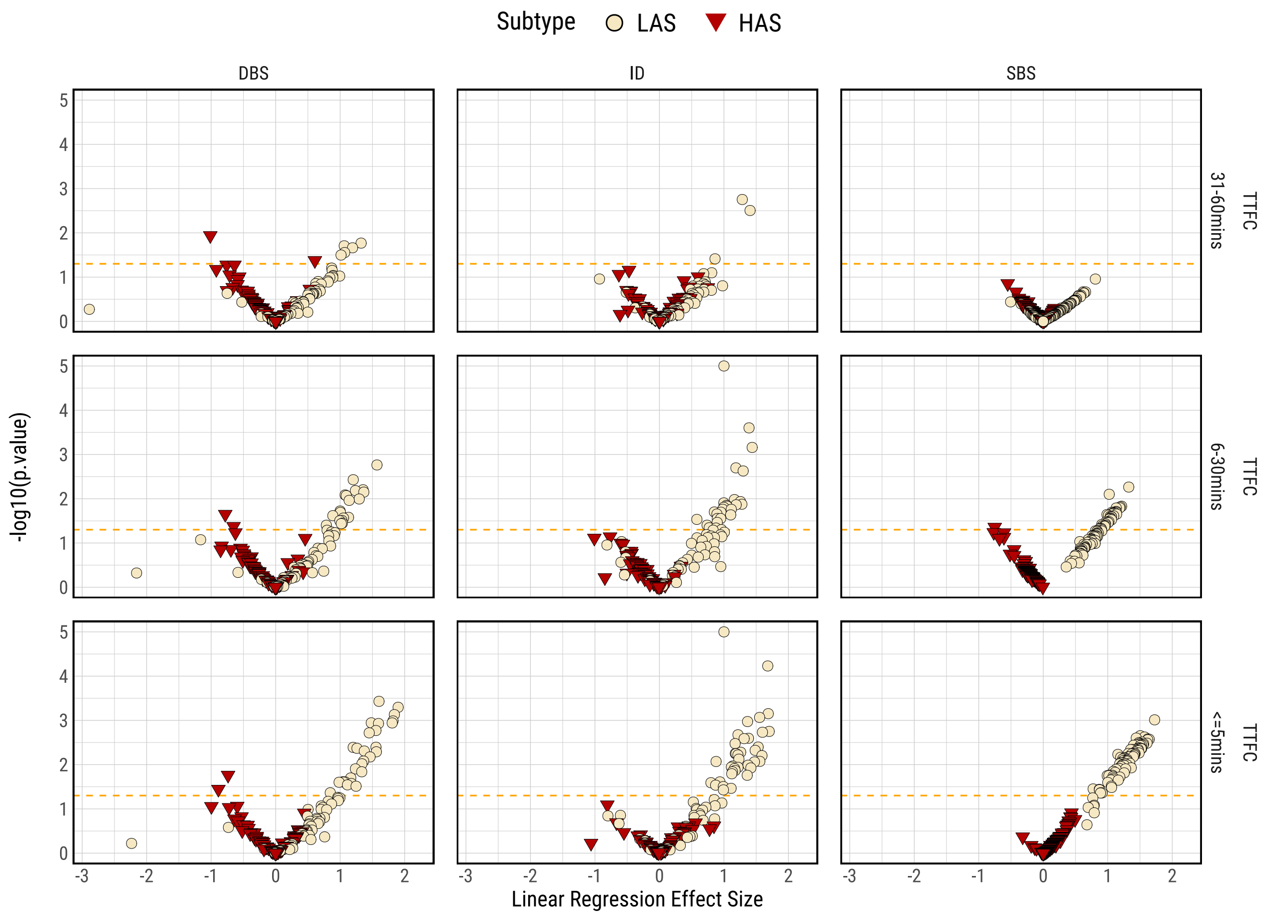

Supplementary Fig. 14

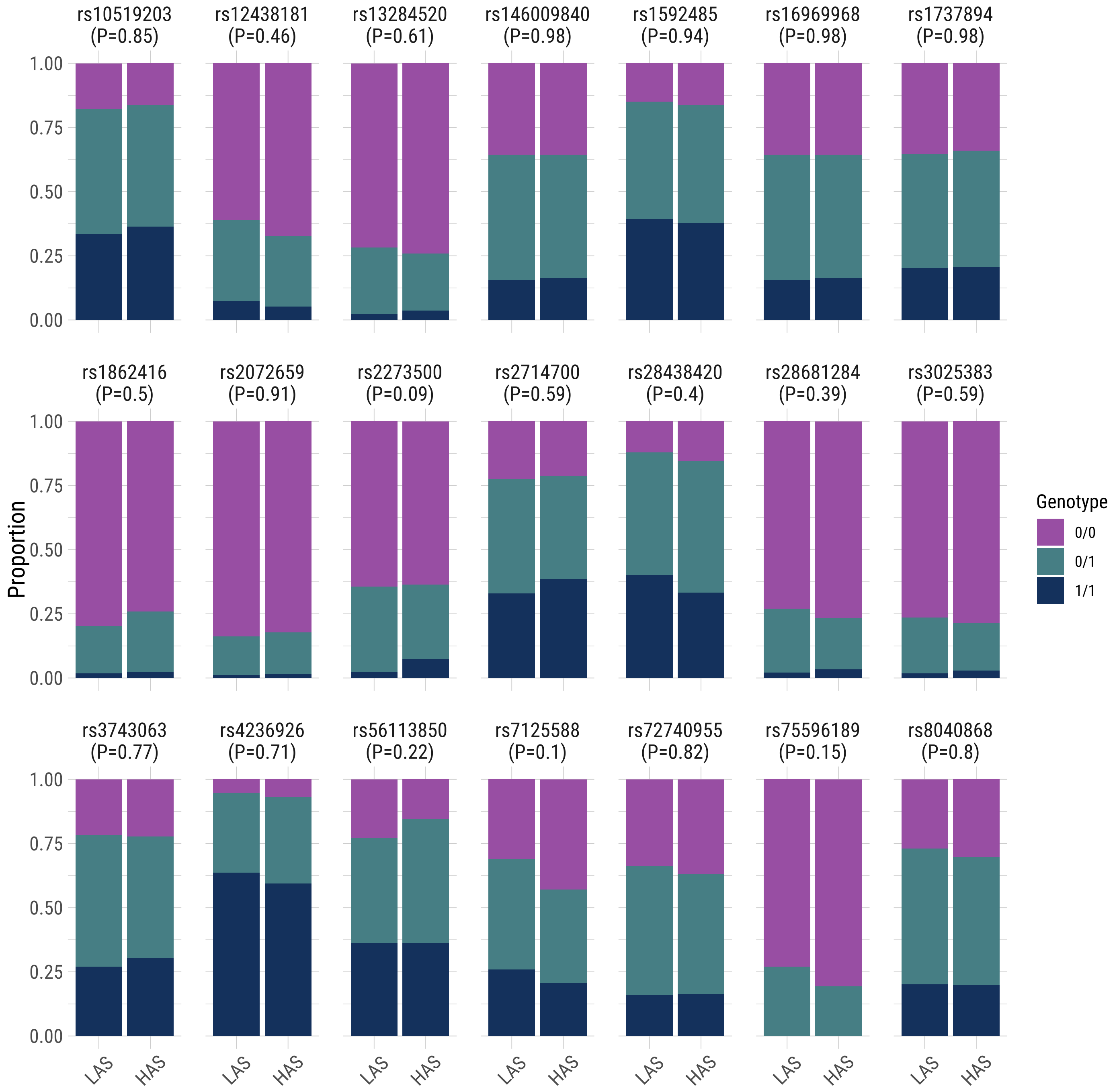

Supplementary Fig. 15

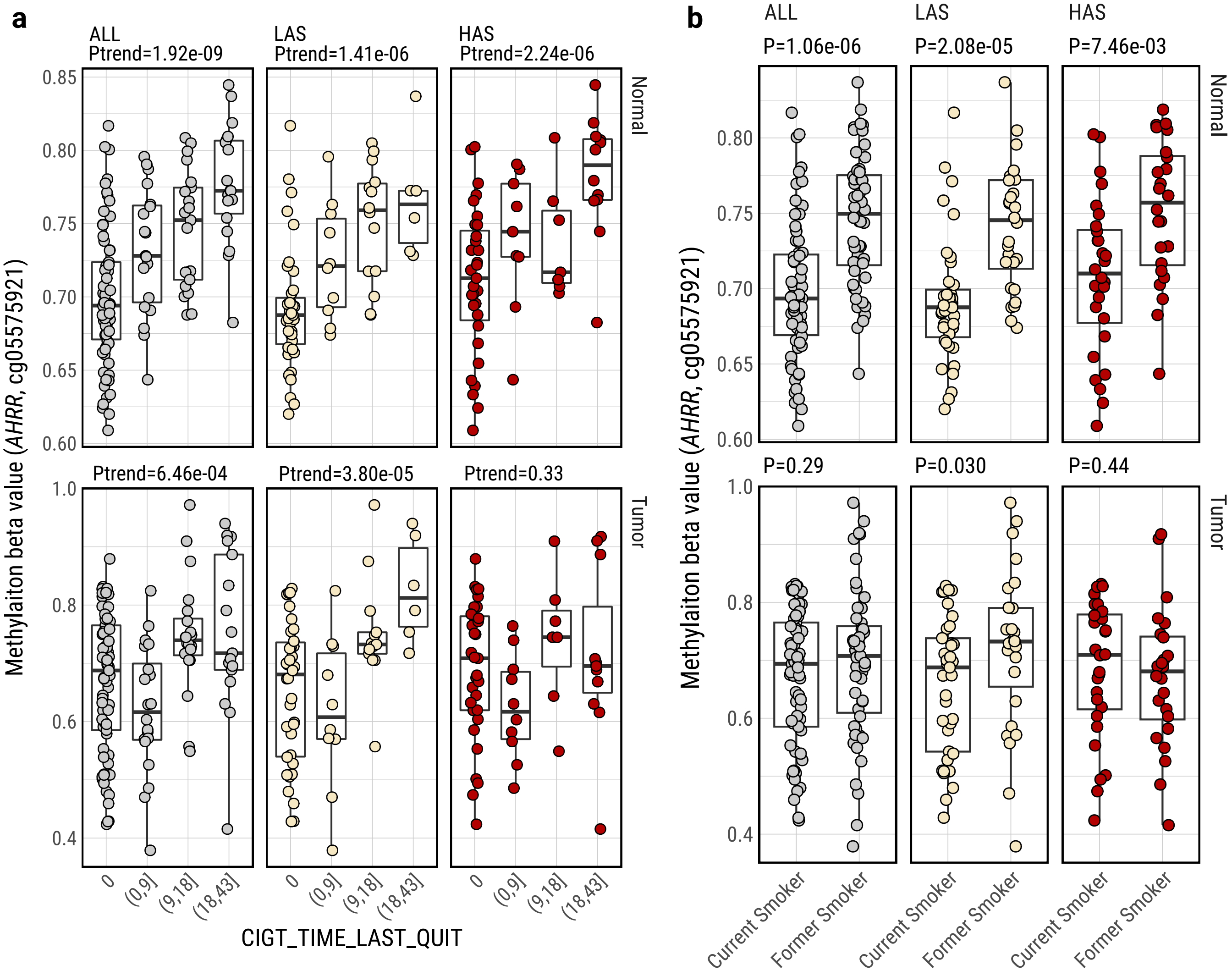

Supplementary Fig. 16

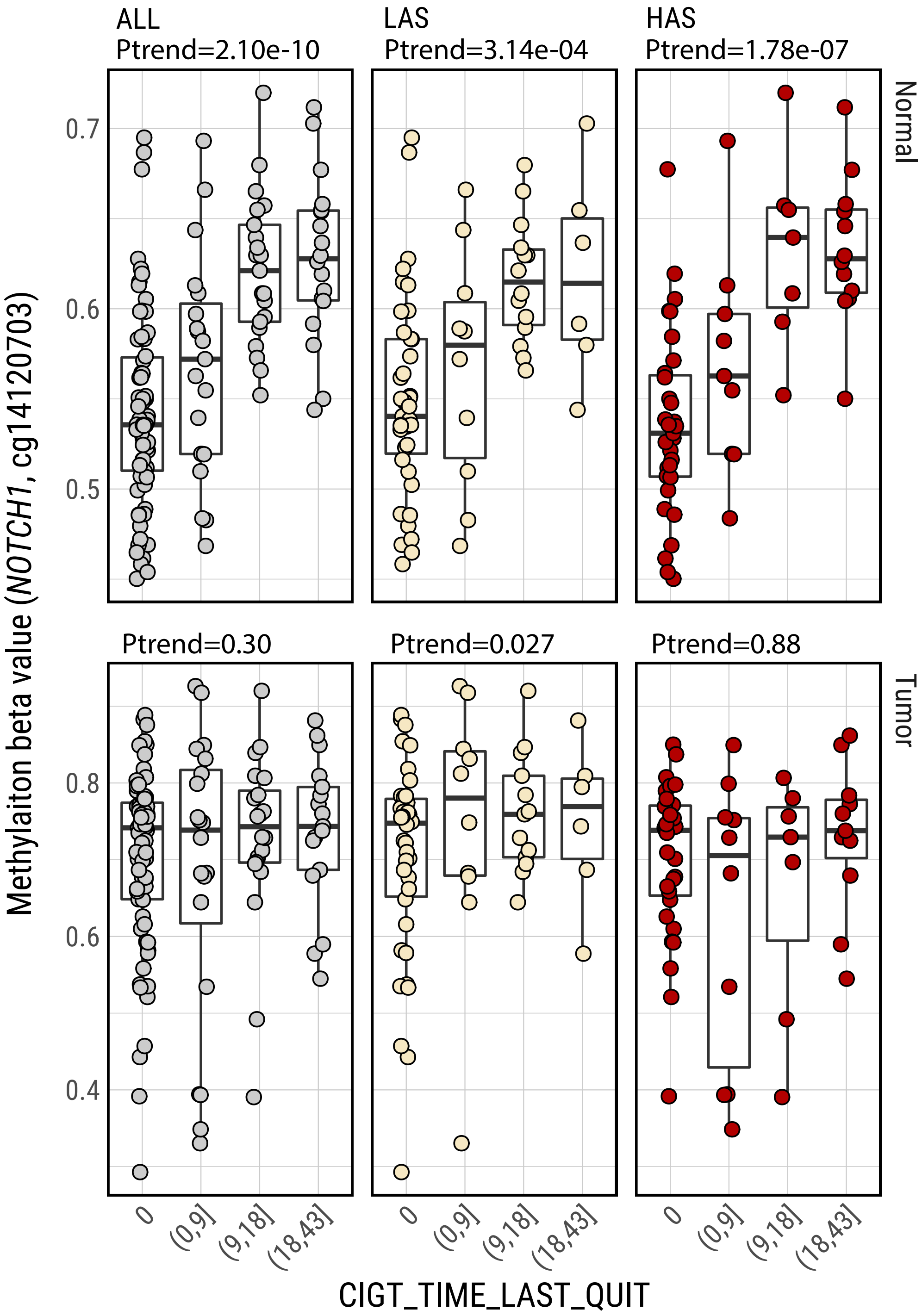

Supplementary Fig. 17

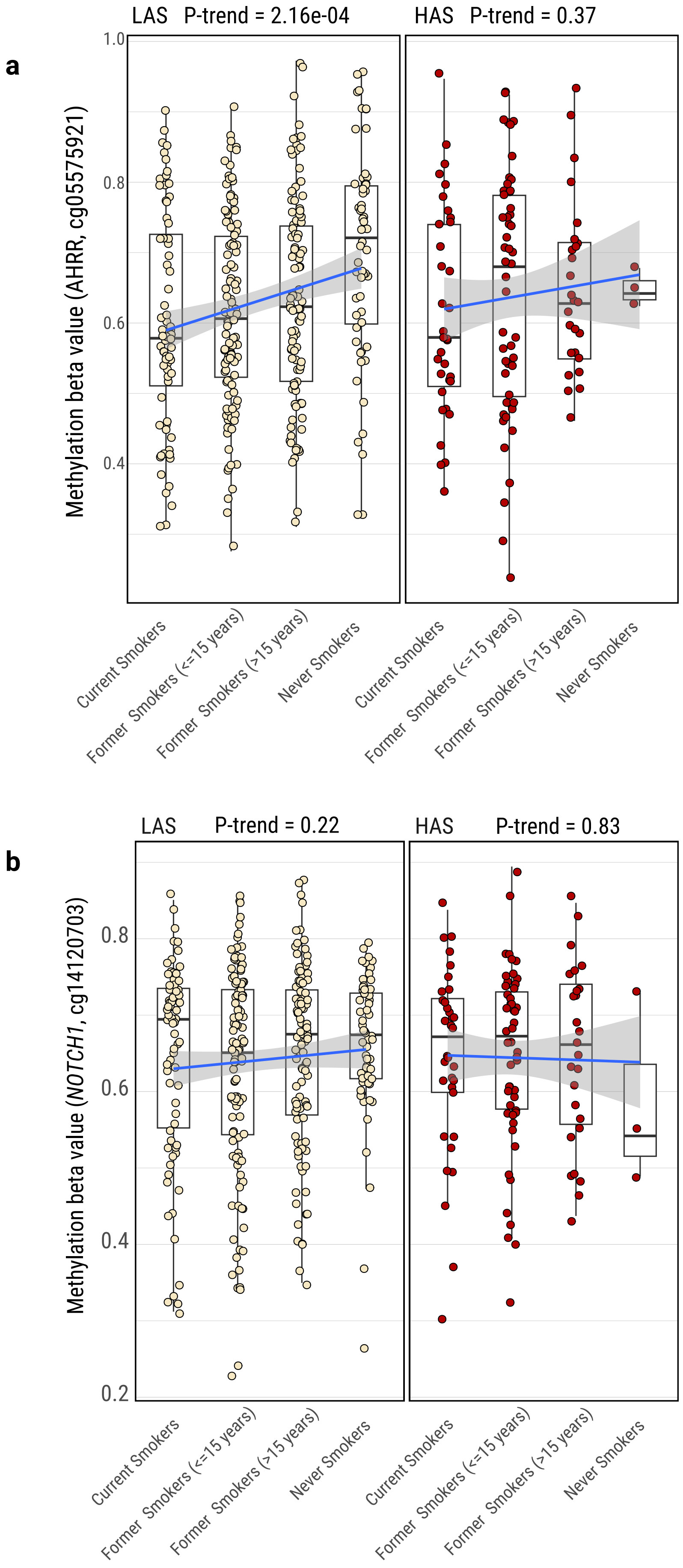

Supplementary Fig. 18

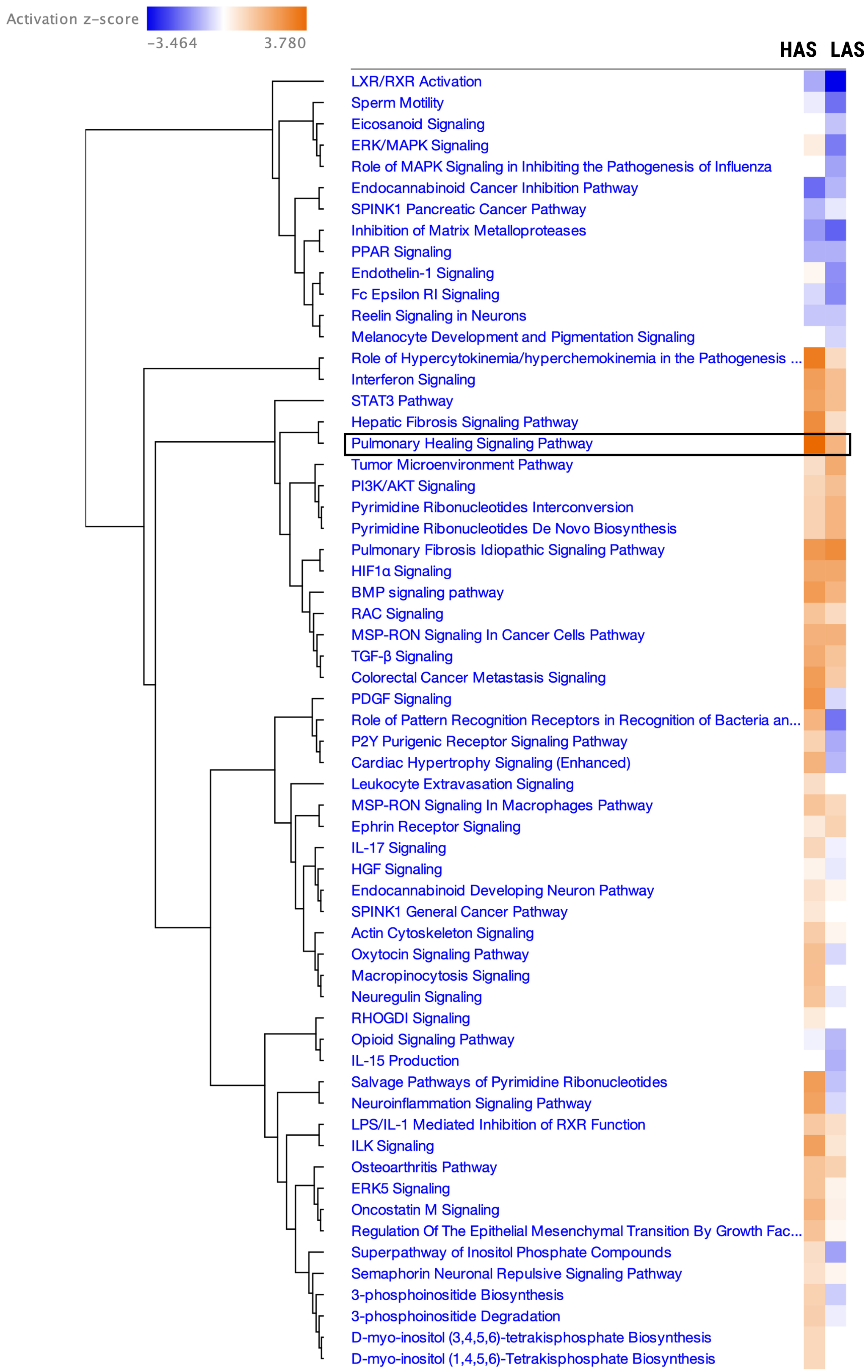

Supplementary Fig. 19

a

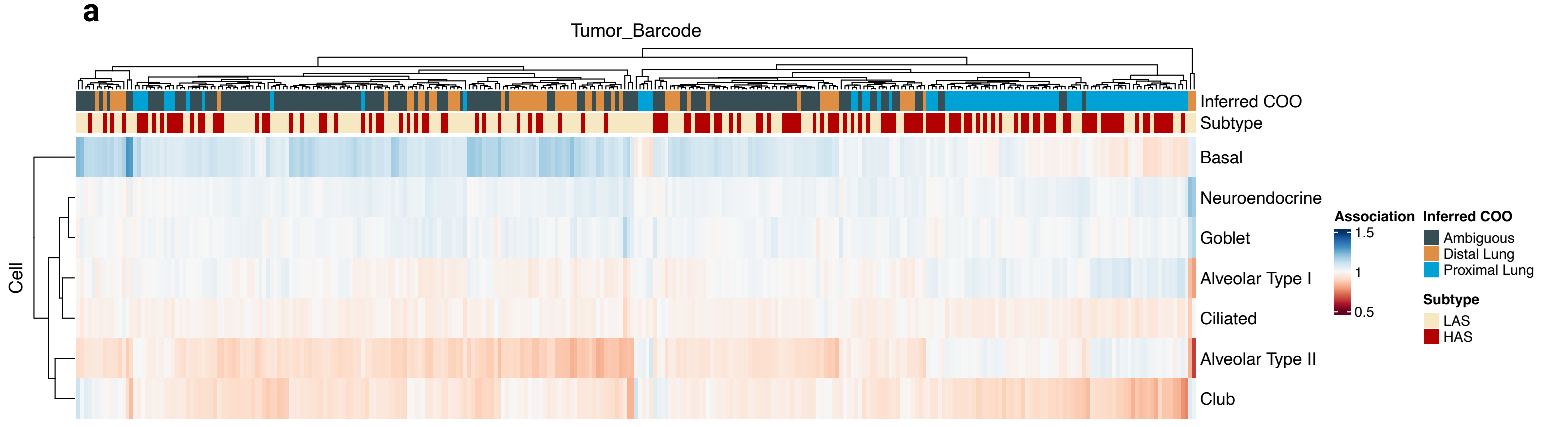

b

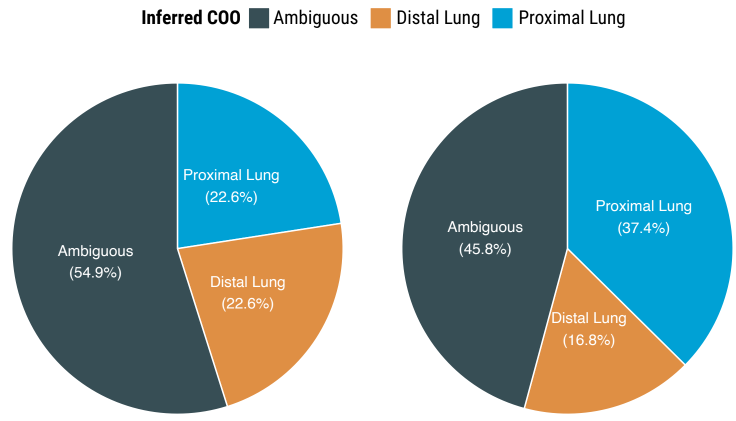

Supplementary Fig. 20

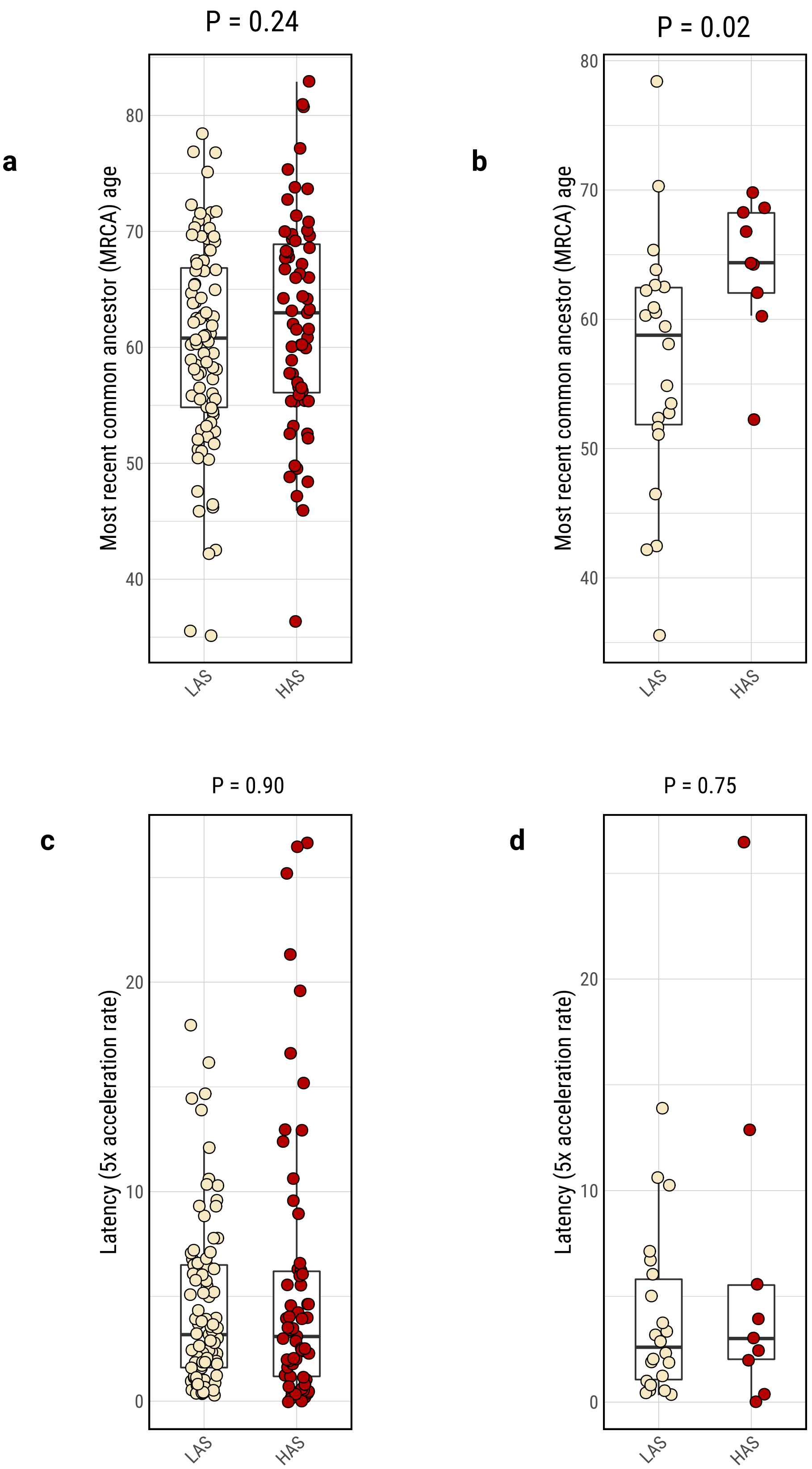

Supplementary Fig. 21

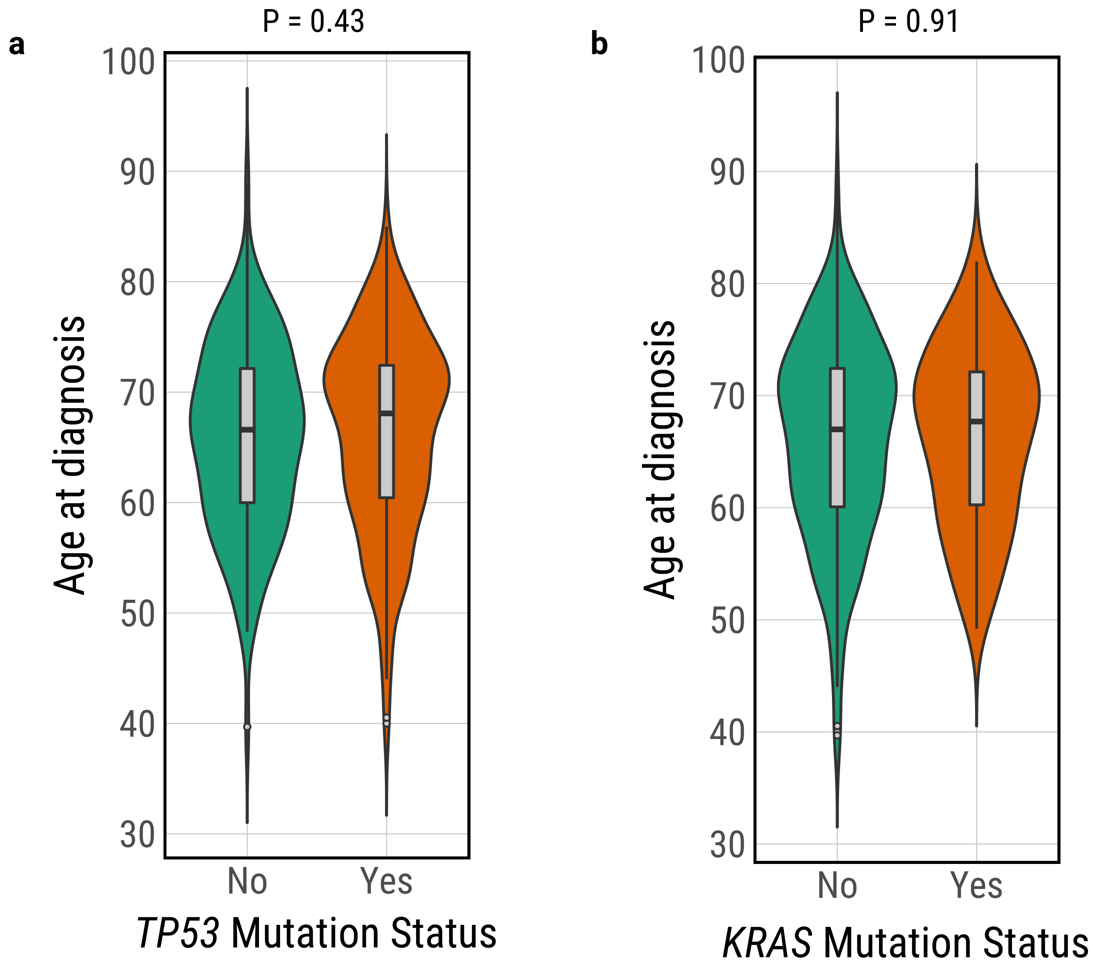

Supplementary Fig. 22

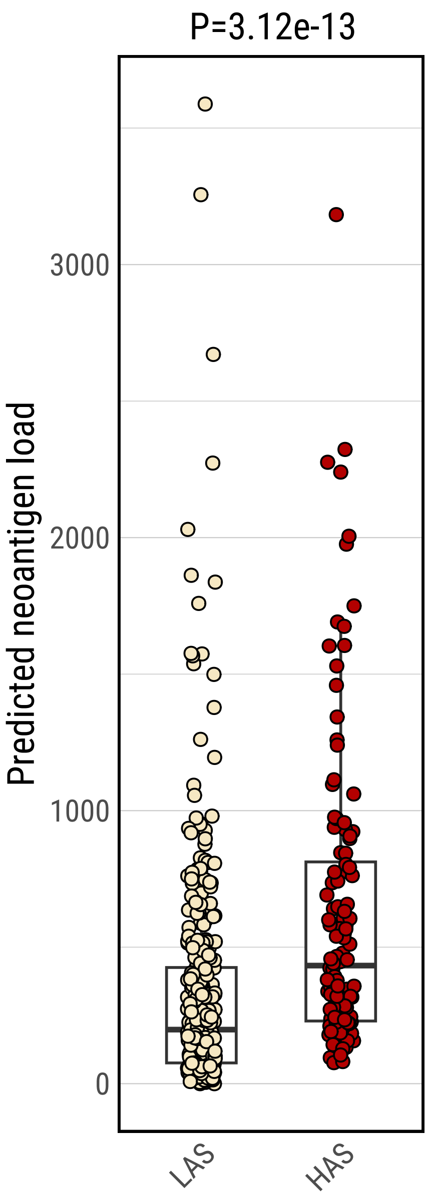

Supplementary Fig. 23

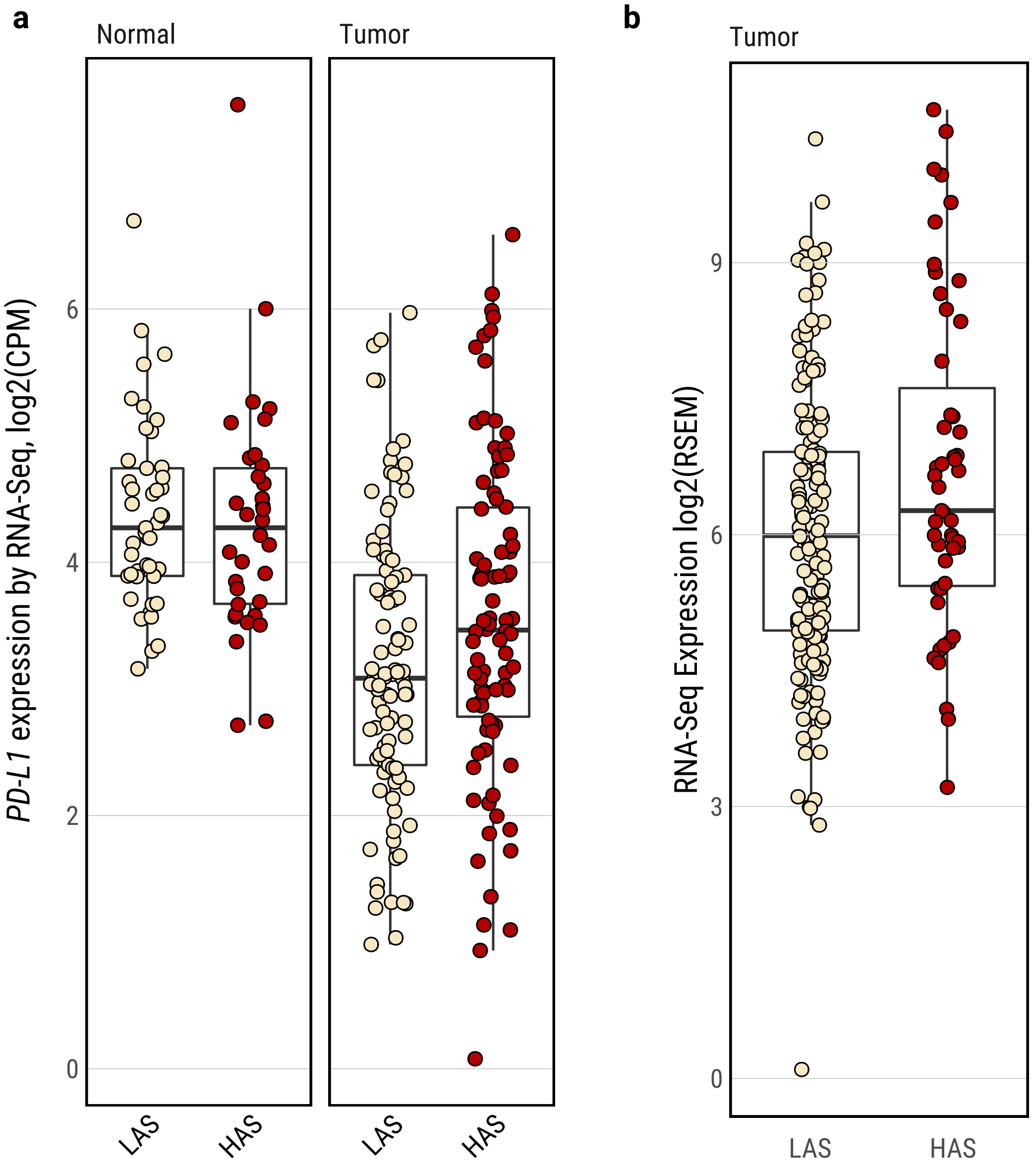

Supplementary Fig. 24

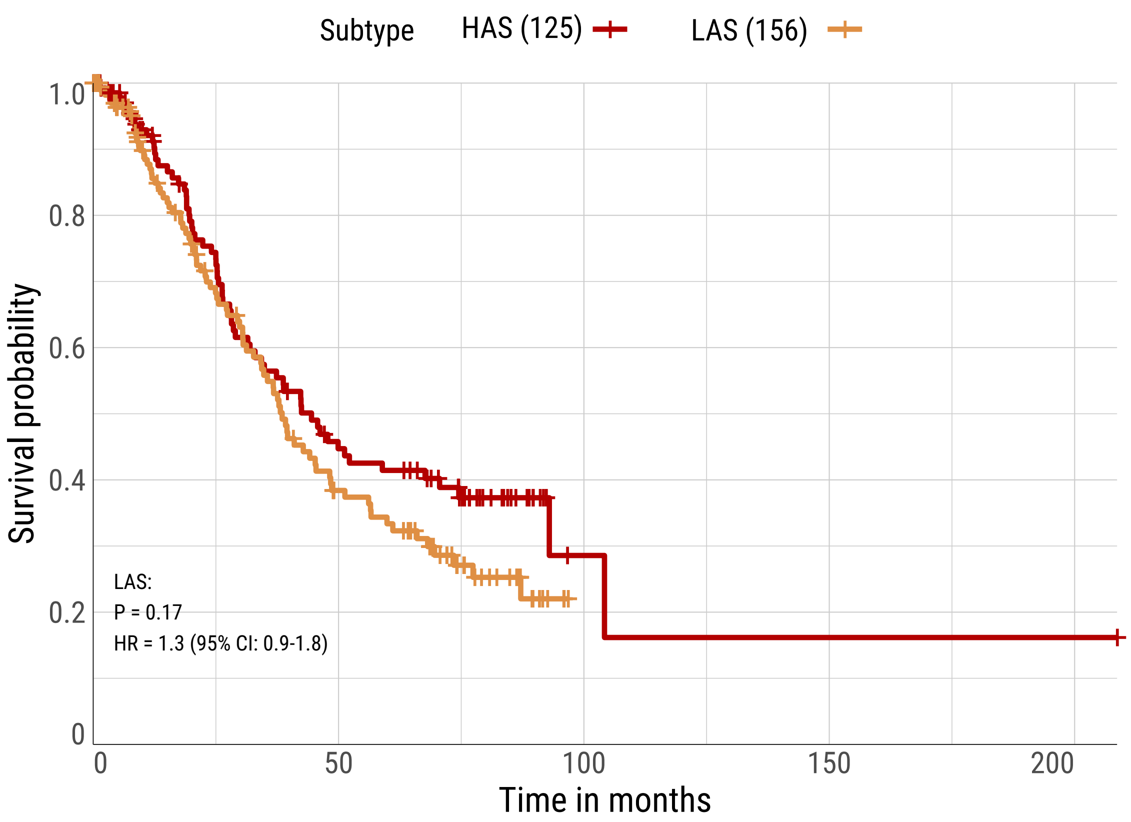

Supplementary Fig. 25

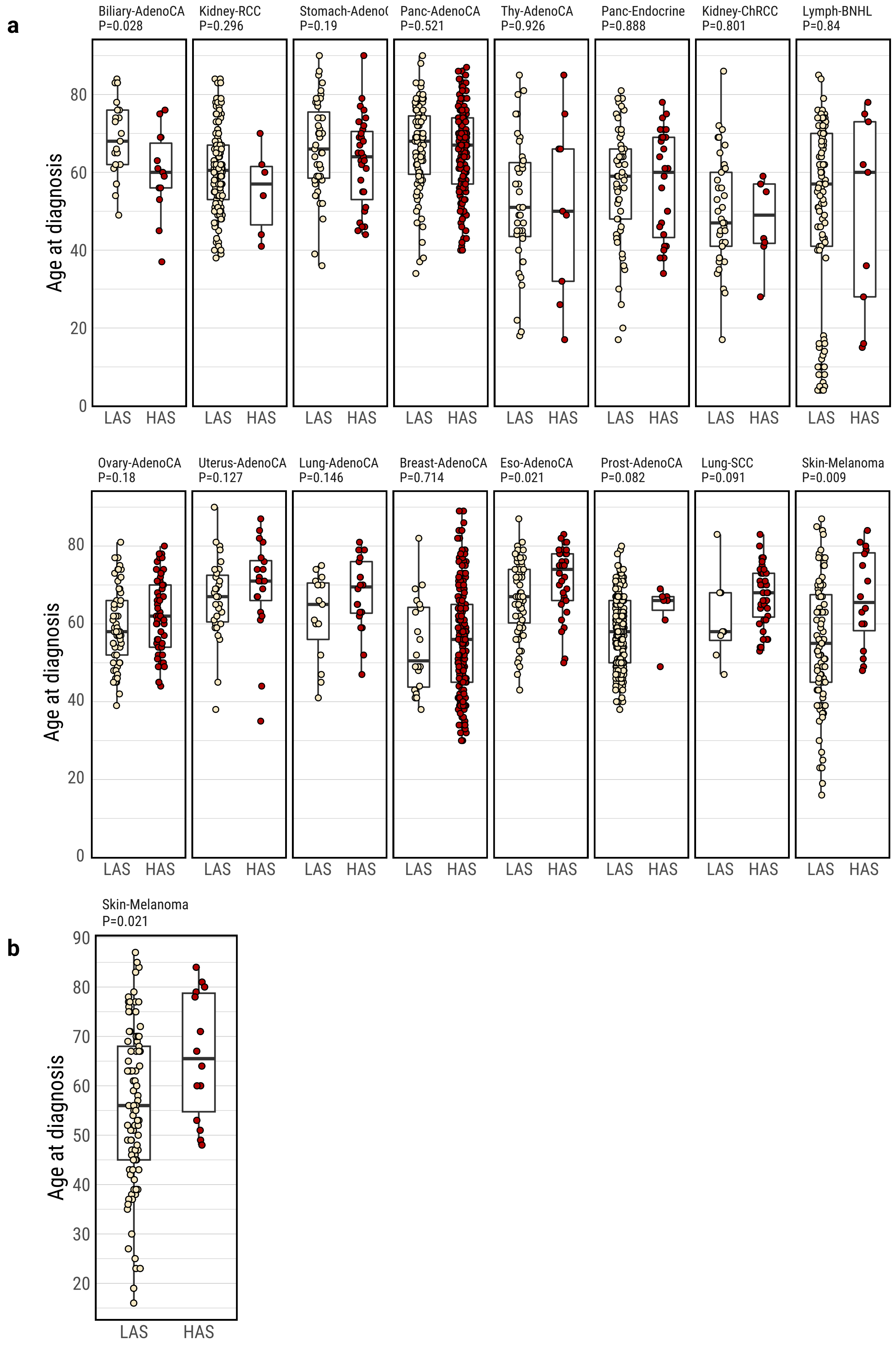

Supplementary Fig. 26

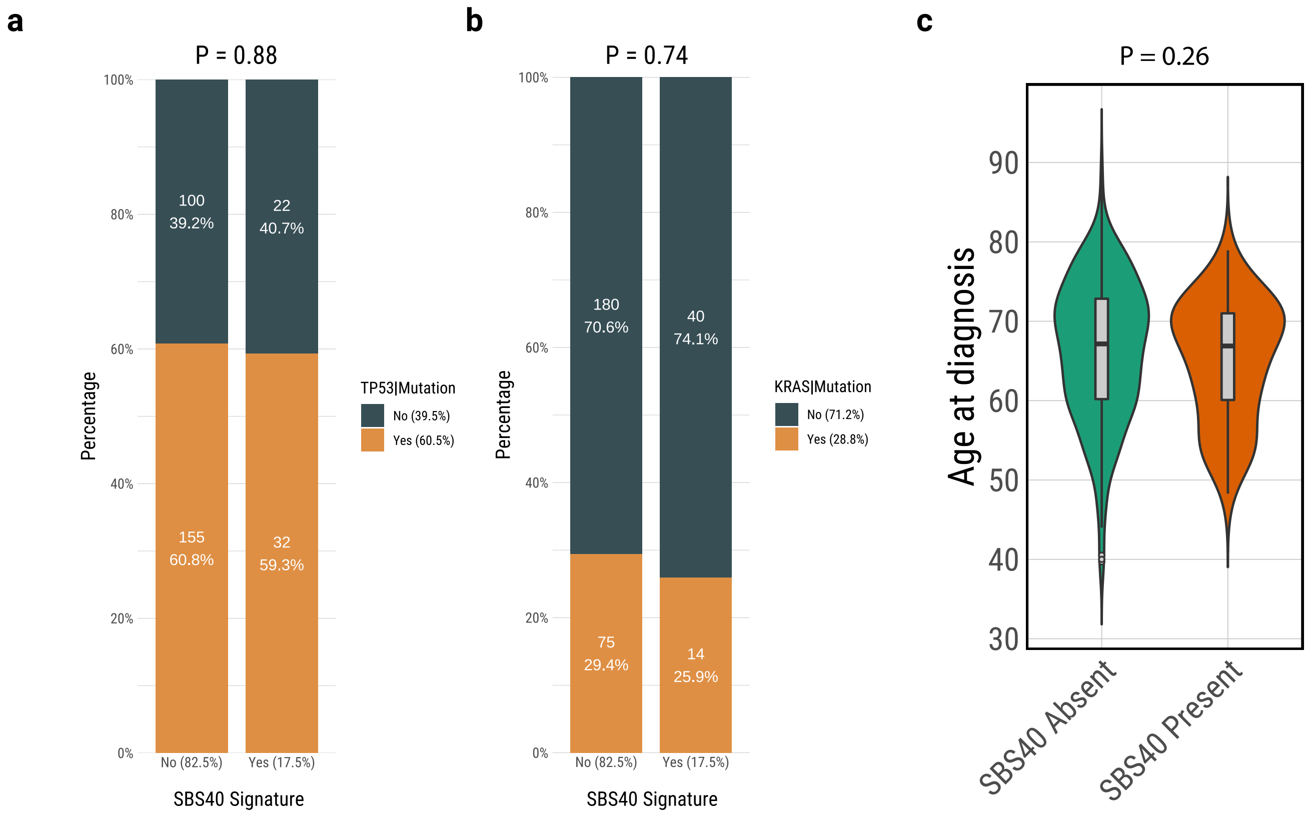

Supplement: Supplement 2 — Supplementary Fig. 1: Summary of multi-omics data and clinical information in this study. Supplementary Fig. 2: Landscape of mutational processes for DBS (a) and ID (b). The landscape of mutational signature plots include a bar plot presenting the total number of mutations assigned to each signature, the proportion plot of signatures assigned to each sample, and cosine similarity between the original mutation profile and signature decomposition. Supplementary Fig. 3: a-d, Comparison of genomic alterations (a), tobacco smoking exposure (b), clinical information (c), and germline APOBEC3B deletion (d) between LAS and HAS tumors. The P-values derived from the Wilcoxon sum rank test or Chi-squared test are shown above the plots. Supplementary Fig. 4: Genomic characterizations of LAS and HAS tumors. The subplots from top to bottom: distribution of genomic alteration numbers, most frequently mutated or potential driver genes, oncogenic fusions, significant focal SCNA, and different genomic features. The numbers on the right of each subplot show the overall frequency or median values. Supplementary Fig. 5: a,b, Logistic regression analysis between tumor subtypes and driver mutation status of driver genes, adjusting for the following covariates: age, sex, histology, TMB, and tumor purity. The significance thresholds P<0.05 (red) and FDR<0.05 (green) are indicated by the dashed lines. Supplementary Fig. 6: Differentially expressed UNG between LAS and HAS tumors based on all available tumors (a) or only in tumors with copy-neutral status for the UNG genomic location (b). Significant P-values from the linear regression are labeled below each boxplot. The linear regression model was adjusted for the following covariates: tumor purity and copy number status in (a) and tumor purity only in (b). Supplementary Fig. 7: Gene expression correlation between UNG and APOBEC3A or APOBEC3B in normal samples from this study (a), tumor samples from TCGA LUAD (b), and normal samples from TCGA [file media-2.pdf]
